# The histone H3K9 methyltransferase SUV39H links SIRT1 repression to myocardial infarction

**DOI:** 10.1038/ncomms14941

**Published:** 2017-03-31

**Authors:** Guang Yang, Xinyu Weng, Yuhao Zhao, Xinjian Zhang, Yuanping Hu, Xin Dai, Peng Liang, Peng Wang, LeiLei Ma, Xiaolei Sun, Lei Hou, Huihui Xu, Mingming Fang, Yuehua Li, Thomas Jenuwein, Yong Xu, Aijun Sun

**Affiliations:** 1 Key Laboratory of Cardiovascular Disease and Molecular Intervention and Key Laboratory of Human Functional Genomics of Jiangsu Province, Department of Pathophysiology, Nanjing Medical University, Nanjing 211166, China; 2 Shanghai Institute of Cardiovascular Disease, Zhongshan Hospital, Fudan University, Shanghai 200032, China; 3 Institute of Biomedical Sciences, Fudan University, Shanghai 200032, China; 4 Department of Nursing, Jiangsu Jiankang Vocational College, Nanjing 210029, china; 5 Department of Epigenetics, Max Planck Institute of Immunobiology and Epigenetics, Stübeweg 51, 79108 Freiburg, Germany

## Abstract

Myocardial infarction (MI) dampens heart function and poses a great health risk. The class III deacetylase sirtuin 1 (SIRT1) is known to confer cardioprotection. SIRT1 expression is downregulated in the heart by a number of stress stimuli that collectively drive the pathogenesis of MI, although the underlying mechanism remains largely obscure. Here we show that in primary rat neonatal ventricular myocytes (NRVMs), ischaemic or oxidative stress leads to a rapid upregulation of SUV39H, the mammalian histone H3K9 methyltransferase, paralleling SIRT1 downregulation. Compared to wild-type littermates, SUV39H knockout mice are protected from MI. Likewise, suppression of SUV39H activity with chaetocin attenuates cardiac injury following MI. Mechanistically, SUV39H cooperates with heterochromatin protein 1 gamma (HP1γ) to catalyse H3K9 trimethylation on the SIRT1 promoter and represses SIRT1 transcription. SUV39H augments intracellular ROS levels in a SIRT1-dependent manner. Our data identify a previously unrecognized role for SUV39H linking SIRT1 trans-repression to myocardial infarction.

## Abstract (web summary)

The molecular pathways regulating the cardioprotective activity of deacetylase sirtuin-1 are unknown. Here, Yang *et al*. show that histone H3K9 methyltransferase SUV39H and HP1gamma cooperatively methylate H3K9 on the sirtuin-1 promoter and inhibit sirtuin-1 transcription, and show that inhibition of SUV39H in mice is cardioprotective.

A number of factors, including senescence, unhealthy dietary choices and life styles, and surges in metabolic disorders, all contribute to the epidemic of ischaemic heart disease (IHD) across a wide spectrum of ages and ethnic groups worldwide. Despite decades of vigorous basic and clinical research, IHD remains the leading cause of death in major industrialized nations^1^. Myocardial infarction (MI), spontaneous or procedural, in patients with IHD, serves to further depress heart function, frequently induces deleterious cardiac remodelling, aggravates organ ischaemia, and is associated with increased risk of heart failure and mortality^2^. Clearly, deciphering the mechanisms that drive the pathogenesis of MI holds the key to stabilizing heart function and reducing MI-related deaths in IHD patients.

During MI pathogenesis, a host of stress stimuli inflict injuries in cardiomyocytes. Ischaemia depletes cellular ATP causing mitochondrial dysfunction and accelerating reactive oxygen species (ROS) generation. Elevated ROS levels, in turn, interfere with oxidative phosphorylation and dampen mitochondrial function creating a self-amplifying vicious cycle to drive the death of cardiomyocytes^3^. By comparison, anti-oxidant therapies have shown varied but promising effects in alleviating MI-induced heart injury in model animals and in IHD patients^4^,^5^. The class III protein deacetylase SIRT1 has been documented to confer cardioprotection in the context of IHD both *in vivo* and *in vitro* at least in part by suppressing ROS generation^6^,^7^,^8^,^9^. In keeping with these observations, SIRT1 expression levels and, by extension, promoter polymorphisms have been found to correlate with acute MI in model animals and in humans^10^,^11^,^12^. The underlying mechanism contributing to altered SIRT1 transcription in ischaemic cardiomyocytes is not understood.

The epigenetic machinery plays a pivotal role in regulating mammalian gene expression. Differential histone modifications represent a prototypical epigenetic mechanism wherein a combination of several modified residues on histone tails barcodes the promoter region and predicts the transcriptional outcome of a specific gene. A simplistic model has been proposed that, confirmed more recently through genome-wide sequencing analyses, trimethylation of histone H3 lysine 4 (H3K4Me3) heralds transcriptional activation, whereas trimethylation of H3 lysine 9 (H3K9Me3) leads to trans-repression. SUV39H, short for suppressor of variegation 3(9) homologue, is the sole enzyme responsible for laying down the H3K9Me3 mark on the chromatin^13^. SUV39H was initially discovered as essential for silencing gene expression in the heterochromatin region. It remains obscure how SUV39H may contribute to gene regulation in the euchromatin region and more importantly, to the pathogenesis of MI.

We report here that upregulation of SUV39H accompanies SIRT1 downregulation in the heart during MI. Genetic deletion or pharmaceutical inhibition of SUV39H ameliorates cardiac injury following MI in mice. SUV39H modulates ROS levels in the heart in a SIRT1-dependent manner. Therefore, SUV39H links SIRT1 trans-repression to myocardial infarction providing rationale for targeting SUV39H1 in the development novel therapeutic strategies to treat ischaemic heart disease.

## Results

### SUV39H levels are upregulated *in vivo* and *in vitro*

**Figure 1:**
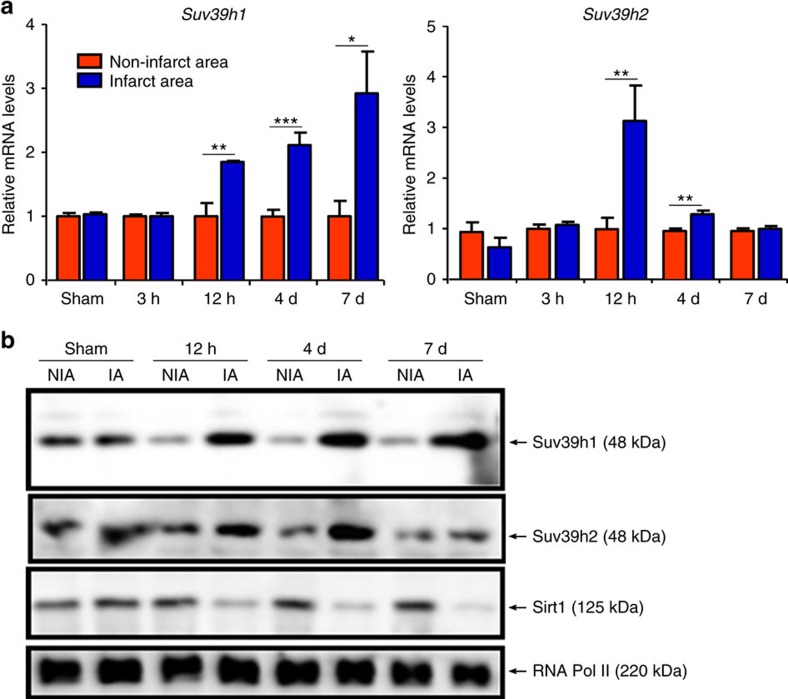
SUV39H levels were upregulated in the heart following MI. (**a**,**b**) MI was induced in C57/BL6 mice by the LAD procedure and the mice were killed at indicated time points. Heart issues from the infarcted area (IA) and the non-infarcted area (NIA) were homogenized and examined for SUV39H and SIRT1 expression by qPCR and western blotting. Error bars represent s.d. (*N*=6 for the sham group and *N*=10 for each of the MI groups). *P<0.05; **P<0.01; ***P<0.001 (one-way ANOVA with *post hoc* Scheffe test). d, days.

We started off by examining the levels of SUV39H in the myocardium following ischaemia challenge in mice. As shown in Fig. 1a, there was a quick increase in *Suv39h1* mRNA levels in ischaemic hearts in mice 12 h after the ligation of left anterior descending (LAD) artery procedure, which was sustained and further augmented at 4 and 7 days. *Suv39h2*mRNA levels were also upregulated in ischaemic hearts at 12 h but receded at 4 and 7 days. Western blotting showed a similar trend in the protein levels of Suv39h1 and Suv39h2 following MI (Fig. 1b). We also examined the expression levels of heterochromatin protein 1 (HP1), the binding partner for SUV39H during heterochromatinzation and gene trans-repression^13^. The Hp1α and Hp1β isoforms were not significantly altered in the heart throughout the observation window of 7 days following MI, but the Hp1γ isoform was upregulated at day 4 and day 7 post MI in ischaemic heart tissues (Supplementary Fig. 1).

**Figure 2:**
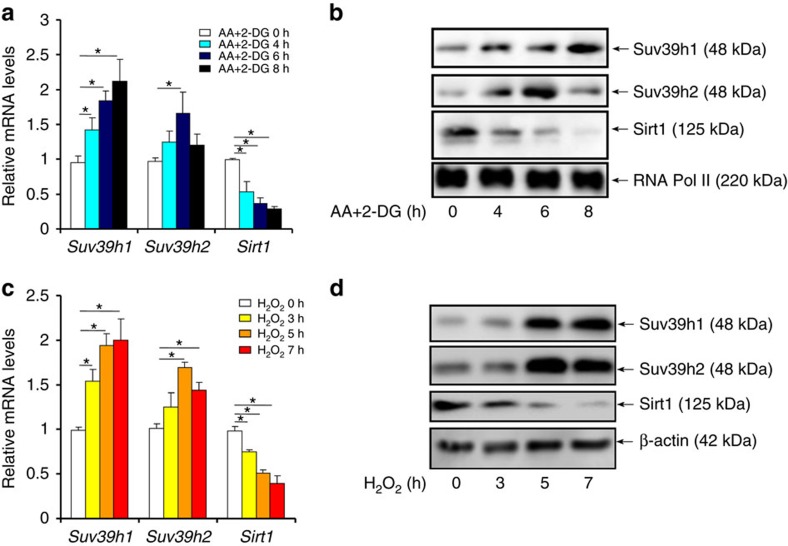
SUV39H were upregulated by ischaemic and oxidative stimuli in cardiomyocytes. (**a**,**b**) Primary rat neonatal ventricular myocytes (NRVMs) were treated with AA+2-DG and harvested at indicated time points. Expression levels were examined by qPCR (**a**) and western blotting (**b**). (**c**,**d**) Primary NRVMs were treated with H_2_O_2_ and harvested at indicated time points. Expression levels were examined by qPCR (**c**) and western blotting (**d**). Error bars represent s.d. (*N*=3). *P<0.05 (one-way ANOVA with *post hoc* Scheffe test).

In primary rat neonatal ventricular myocytes (NRVMs), ischaemic stress (Fig. 2a,b), simulated by the addition of antimycin A plus 2-deoxy glucose (AA+2-DG) to deplete intracellular ATP^14^, and oxidative stress (Fig. 2c,d), induced by the addition of hydrogen peroxide (H_2_O_2_) (refs 15, 16), both stimulated the expression of Suv39h1 and, to a lesser extent, Suv39h2. Similar observations were made in an immortalized rat myoblast cell line H9C2 (Supplementary Fig. 2). Taken together, we conclude that SUV39H levels are responsive to MI-relevant stimuli in the heart and cultured cardiomyocytes indicative of a potential role for SUV39H in MI pathogenesis.

### SUV39H deficiency protects against MI in mice

**Figure 3:**
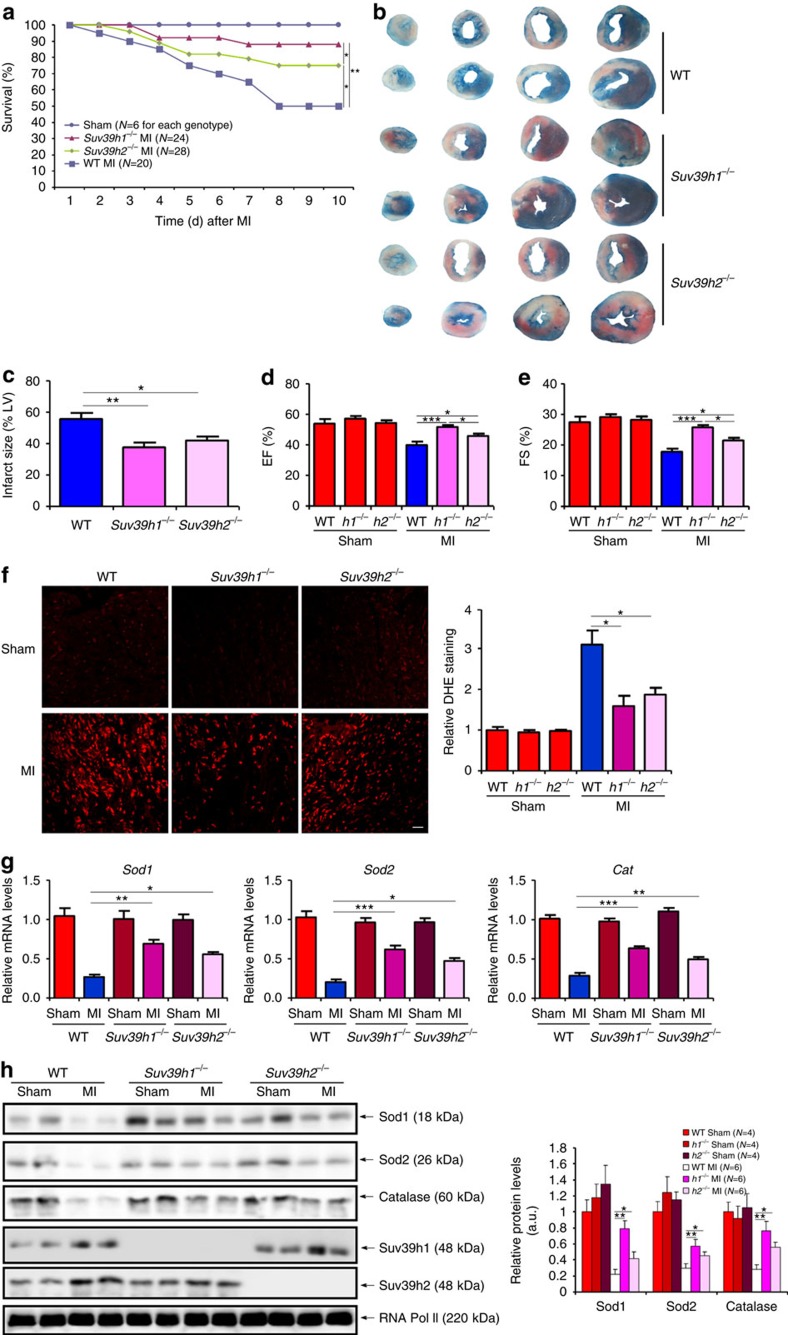
SUV39H deficiency protects against MI in mice. MI was induced in Suv39h1 knockout (*h1*^−/−^) mice, Suv39h2 knockout (*h2*^−/−^) mice or wide-type (WT) littermates by LAD. (**a**) Kaplan–Meier plot showing the survival rates up to 7 days after MI. (**b**) Representative TTC staining. (**c**) Infarct size was calculated and quantified by Image Pro. Error bars represent s.d (*N*=6 for the sham group and *N*=10 for each of the MI groups). (**d**,**e**) EF and FS values were measured by echocardiography. Error bars represent s.d. (*N*=4 for the sham group and *N*=8 for each of the MI groups). (**f**) Cardiac ROS levels were evaluated by DHE staining. Scale bar, 50 μm. Error bars represent s.d. (*N*=3 for the sham group and *N*=6 for each of the MI groups). (**g**,**h**) Expression levels of anti-oxidant genes were measured by qPCR (**g**) and western blotting (**h**). Error bars represent s.d. (*N*=4 for the sham group and *N*=8 for each of the MI groups). *P<0.05; **P<0.01 (one-way ANOVA with *post hoc* Scheffe test).

To assign a role for SUV39H in MI pathogenesis, we performed the following experiments. MI was induced in *Suv39h1* knockout (*Suv39h1*^−/−^) mice, *Suv39h2* knockout (*Suv39h2*^−/−^) mice or wide-type (WT) littermates by the LAD procedure. Kaplan–Meier analysis indicated that both *Suv39h1*^−/−^ and *Suv39h2*^−/−^ mice had a significant advantage of survival over WT mice post MI; additionally, *Suv39h1*^−/−^ mice survived significantly longer than *Suv39h2*^−/−^ mice (Fig. 3a). Post-mortem autopsy revealed that while both *Suv39h1*^−/−^ and *Suv39h2*^−/−^ mice had fewer incidents of cardiac rupture than WT mice, *Suv39h1*^−/−^ mice were less prone to cardiac rupture than *Suv39h2*^−/−^ mice (Supplementary Table 1). Quantitative PCR and western blotting indicated that matrix metalloproteinase 9 (MMP9) induction following MI was much more prominent in WT mice than in either *Suv39h1*^−/−^ mice or *Suv39h2*^−/−^ mice (Supplementary Fig. 3). Indeed, both *Suv39h1*^−/−^ mice and *Suv39h2*^−/−^ mice had a smaller infarct size than WT littermates (Fig. 3b,c). TUNEL assay provided corroborating evidence, which suggested that there was more extensive cardiomyocyte death in WT mice as compared to *Suv39h1*^−/−^ mice or *Suv39h2*^−/−^ mice (Supplementary Fig. 4). These observations probably explained why *Suv39h1*^−/−^ and *Suv39h2*^−/−^ mice exhibited better heart functions as evidenced by echocardiographic assessment of EF (Fig. 3d) and FS (Fig. 3e) values.

Since deficient ROS elimination plays a role in MI pathogenesis, we evaluated the effect of SUV39H deficiencies on ROS levels *in vivo*. As shown in Fig. 3f, dihydroethidium (DHE) staining demonstrated that ROS accumulation was much stronger in WT hearts than either *Suv39h1*^−/−^ or *Suv39h2*^−/−^ hearts. Furthermore, SUV39H deficiencies significantly attenuated the down-regulation of several anti-oxidant genes including superoxide dismutase 1 (*Sod1*), superoxide dismutase 2 (*Sod2*) and catalase (*Cat*) in the myocardium following MI (Fig. 3g,h).

To probe a myeloid lineage-specific role for SUV39H in myocardial infarction, we performed bone marrow transplantation assay. To this end, MI was induced in wild-type mice receiving bone marrow from either wild-type, *Suv39h1*^−/−^ or *Suv39h2*^−/−^ mice. Post-mortem autopsy revealed that there was no significant difference in terms of infarct size; nor was there any improvement in post-MI heart function (Supplementary Fig. 5). Consistently, expression levels of anti-oxidant genes and cardiac ROS levels were not significantly altered among three different chimeric mice (Supplementary Fig. 6). Taken together, these data strongly support a role for SUV39H in promoting MI in mice.

### SUV39H inhibition attenuates MI in mice

**Figure 4:**
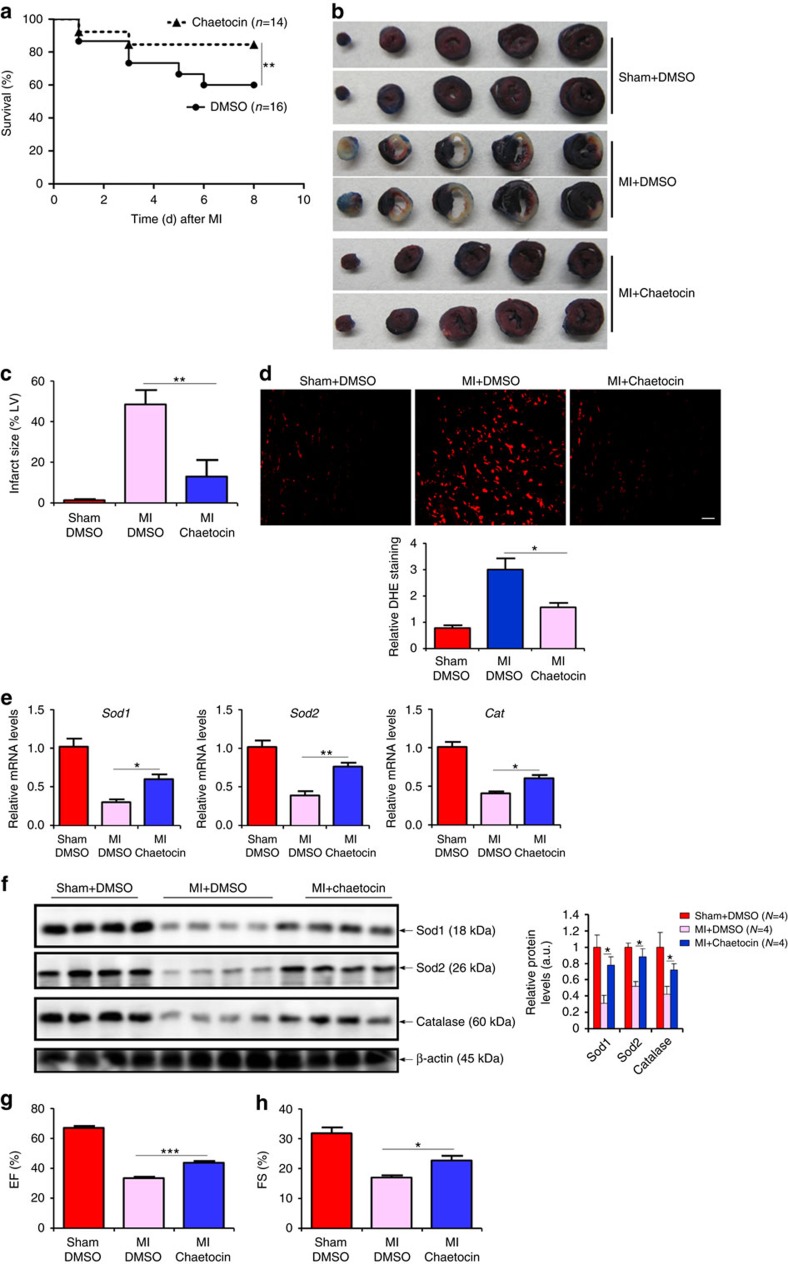
SUV39H inhibition attenuates MI in mice. C57/BL mice were injected peritoneally with chaetocin (25 mg kg^−1^) or DMSO 2 days prior to the LAD procedure. (**a**) Kaplan–Meier plot showing the survival rates up to 7 days after MI. (**b**) Representative TTC staining. (**c**) Infarct size was calculated and quantified by Image Pro. Error bars represent s.d. (*N*=8 for the sham group and *N*=10 for each of the MI groups). (**d**) Cardiac ROS levels were evaluated by DHE staining. Scale bar, 50 μm. Error bars represent s.d. (*N*=3 for the sham group and *N*=6 for each of the MI groups). (**e**,**f**) Expression levels of anti-oxidant genes were measured by qPCR (**g**) and western blotting (**h**). Error bars represent s.d. (*N*=4 for the sham group and *N*=6 for each of the MI groups). (**g**,**h**) EF and FS values were measured by echocardiography. Error bars represent s.d. (*N*=4 for the sham group and *N*=8 for each of the MI groups). *P<0.05; **P<0.01; ***P<0.001 (one-way ANOVA with *post hoc* Scheffe test).

Next, we sought additional evidence that SUV39H might play a role in the pathogenesis of MI. To this end, male C57/BL6 mice were injected peritoneally with chaetocin, a reported inhibitor of SUV39H activity^17^, or DMSO prior to the LAD procedure. Histone methyltransferase assays performed using heart tissue homogenates indicated that chaetocin injection markedly inhibited the activities of both SUV39H1 and SUV39H2 in mice (Supplementary Fig. 7). Chaetocin administration significantly improved the survival rate of mice (Fig. 4a) and reduced infarct size (Fig. 4b,c). Accompanying these changes there was a reduction in *Mmp9* expression, as measured by qPCR and western blotting (Supplementary Fig. 8A,B), and cardiomyocyte apoptosis, as measured by TUNEL assay (Supplementary Fig. 8C), in the heart following MI. DHE staining showed that cardiac ROS levels were suppressed by chaetocin administration (Fig. 4d). Furthermore, chaetocin administration was able to rescue the repression of anti-oxidant genes by MI (Fig. 4e,f). These protective effects afforded by chaetocin cumulatively contributed to improved post-MI heart function (Fig. 4g,h).

We also evaluated the effect of chaetocin on cardiac remodelling 4 weeks after infarction. Picrosirius red and Masson's trichrome staining showed that chaetocin ameliorated interstitial fibrosis (Supplementary Fig. 9A). qPCR and western blotting measurements confirmed that chaetocin pre-empted the induction of pro-fibrogenic genes including type I collagen and alpha smooth muscle actin (α-SMA) in MI hearts (Supplementary Fig. 9B,C). Collectively, these data argue that SUV39H inhibition could not only reduce infarction but also block adverse cardiac remodelling in mice.

### SUV39H represses SIRT1 transcription

**Figure 5:**
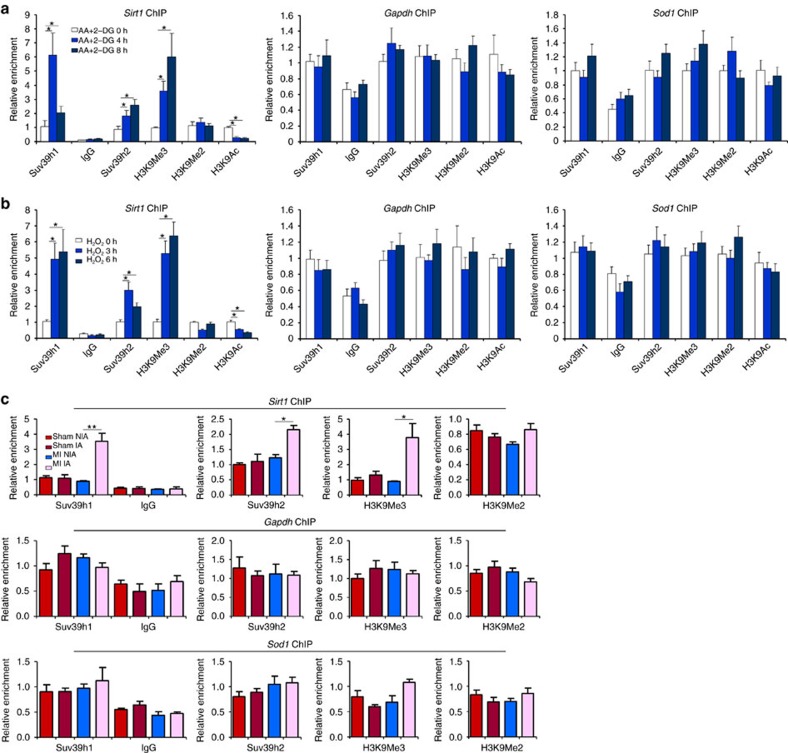
SUV39H represses SIRT1 transcription. (**a**,**b**) Primary NRVMs were treated with AA+2-DG (**a**) or H_2_O_2_ (**b**). ChIP assays were performed with indicated antibodies. (**c**) ChIP assays were performed with heart homogenates from MI mice or sham mice. Error bars represent s.d. (*N*=3 for each group). *P<0.05; **P<0.01 (one-way ANOVA with *post hoc* Scheffe test).

Next, we tackled the mechanism whereby SUV39H may contribute to MI pathogenesis. SIRT1 has been assigned a cardiac-protective role and its expression is inversely correlated with acute MI in humans. Of interest, we observed an inverse correlation between SUV39H expression and SIRT1 expression in mice (Fig. 1). Thus, we proposed that SUV39H might promote MI by targeting SIRT1. To verify this hypothesis, we performed the following experiments. Reporter assays (Supplementary Fig. 10A) showed that SUV39H1 was able to repress the activity of the shortest SIRT1 promoter (−115/+54). In a parallel experiment, we found that ecotopic (Myc-tagged) SUV39H could indeed bind to and consequently stimulate the accumulation of trimethylated H3K9 on the reporter construct transfected into H9C2 cells (Supplementary Fig. 10B). Chromatin immunoprecipitation (ChIP) assay showed that following AA+2-DG stimulation, SUV39H1 quickly assembled on this region of the *Sirt1* promoter but not the *Gapdh* promoter in both NRVMs (Fig. 5a) and H9C2 cells (Supplementary Fig. 11A); by comparison, SUV39H2 was recruited with much weaker affinity. Of interest, there was no significant enrichment of either SUV39H1 or SUV39H2 on the *Sod1* promoter despite strong trans-repression by AA+2-DG treatment, indicating that SUV39H might regulate *Sod1* transcription indirectly. Paralleling SUV39H binding, trimethylated histone H3K9 started to accumulate on the *Sirt1* promoter while acetylated H3K9, an antagonistic marker to H3K9Me3, departed from the *Sirt1* promoter consistent with its trans-repression. Similar observations were made with H_2_O_2_ treatment (Fig. 5b, Supplementary Fig. 11B). We did not detect significant enrichment of H3K9Me2, another prominent histone modification associated with repressed chromatin, on the *Sirt1* promoter suggesting that H3K9Me2 might not play a role in SIRT1 trans-repression in the context of MI. Unlike HP1α and HP1β, HP1γ exhibited enhanced binding to the *Sirt1* promoter (but not to the *Gapdh* promoter or the *Sod1* promoter) in NRVMs treated with either AA+2-DG or H_2_O_2_ (Supplementary Fig. 12). In keeping with this observation, HP1γ also formed a complex with SUV39H1 and SUV39H2 (albeit with lower affinity than SUV39H1) on the *Sirt1* promoter as evidenced by Re-ChIP assay (Supplementary Fig. 13).

More importantly, we discovered that SUV39H1 and SUV39H2 were recruited to the *Sirt1* promoter and interacted specifically with HP1γ in the infarcted myocardium in mice (Fig. 5c, Supplementary Fig. 14), suggesting that SUV39H probably serves as a common link to SIRT1 trans-repression. Finally, reporter assay confirmed that SUV39H1 not only dose-dependently repressed the *Sirt1* promoter but also enhanced the trans-repression of *Sirt1* promoter by either AA+2-DG (Supplementary Fig. 15A) or H_2_O_2_ (Supplementary Fig. 15B). Again, of the three HP1 isoforms only HP1γ repressed the *Sirt1* promoter in the presence of either AA+2-DG or H_2_O_2_ (Supplementary Fig. 16A–C). Using ChIP assay, we confirmed that ectopically introduced (HA-tagged) HP1γ could indeed occupy the SIRT1 reporter construct in H9C2 cells (Supplementary Fig. 16D). Therefore, we conclude that SUV39H represses *Sirt1* transcription in cardiomyocytes.

### SUV39H silencing or inhibition restores SIRT1 transcription

**Figure 6:**
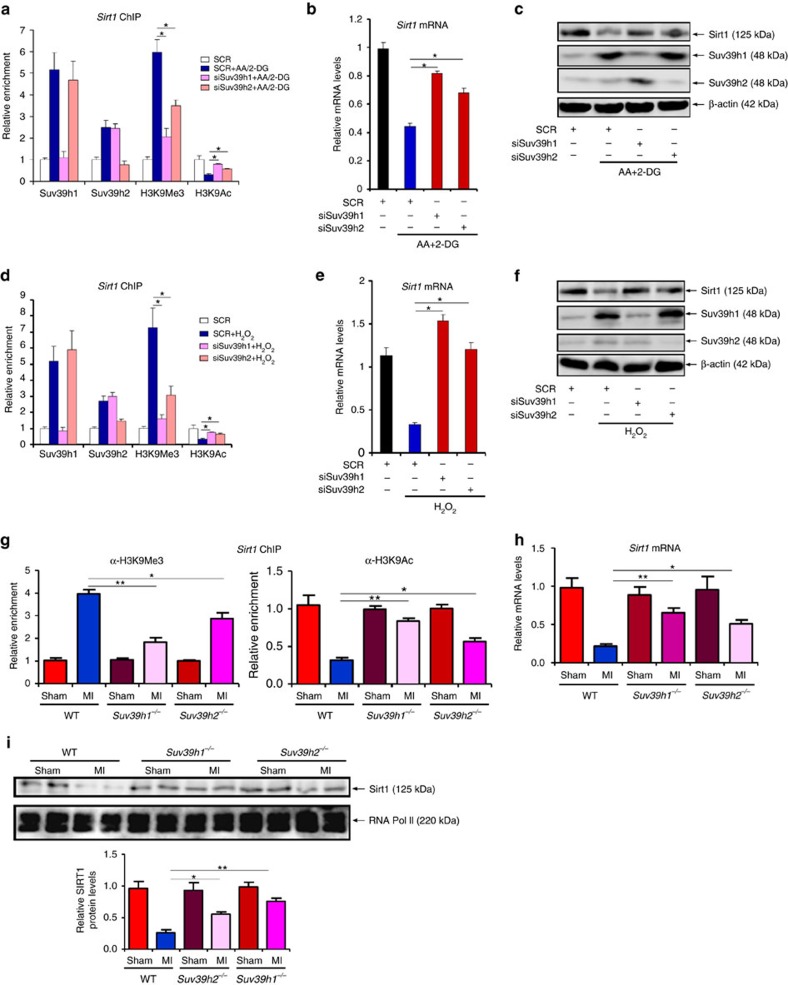
SUV39H silencing restores SIRT1 transcription. (**a**–**c**) Primary NRVMs were transfected with SUV39H siRNA or random siRNA (SCR) followed by treatment with AA+2-DG. ChIP assays were performed with indicated antibodies (**a**). SIRT1 expression was examined by qPCR (**b**) and western blotting (**c**). (**d**–**f**) Primary NRVMs were transfected with SUV39H siRNA or random siRNA (SCR) followed by treatment with H_2_O_2_. ChIP assays were performed with indicated antibodies (**d**). SIRT1 expression was examined by qPCR (**e**) and western blotting (**f**). (**g**–**i**) MI was induced in Suv39h1knockout (*h1*^−/−^) mice, Suv39h2 knockout (*h2*^−/−^) mice or wide-type (WT) littermates by LAD. ChIP assays were performed with indicated antibodies (**g**). SIRT1 expression was examined by qPCR (**h**) and western blotting (**i**). Error bars represent s.d. (*N*=3 for each group). *P<0.05; **P<0.01 (one-way ANOVA with *post hoc* Scheffe test).

We next employed the following strategies to confirm that SUV39H is indispensible for SIRT1 trans-repression in the context of MI. siRNA-mediated knockdown of SUV39H1 or SUV39H2 abrogated H3K9Me3 assembly and restored H3K9Ac levels on the *Sirt1* promoter despite the stimulation by AA+2-DG in cardiomyocytes (Fig. 6a, Supplementary Fig. 17A). Accordingly, SUV39H knockdown normalized *Sirt1* expression at both mRNA (Fig. 6b, Supplementary Fig. 17B) and protein (Fig. 6c, Supplementary Fig. 17C) levels. Likewise, SUV39H depletion also remodelled the chromatin structure surrounding the *Sirt1* promoter (Fig. 6d, Supplementary Fig. 18A) and restored *Sirt1* expression (Fig. 6e,f, Supplementary Fig. 18B,C) in H_2_O_2_-treated cardiomyocytes. In SUV39H1 and SUV39H2 deficient mice, we observed more H3K9Ac and less H3K9Me3 on the *Sirt1* promoter as opposed to WT mice (Fig. 6g), which probably contributed to higher SIRT1 levels in infarcted hearts (Fig. 6h,i). Consistent with the prior observations that HP1γ interacted with SUV39H to repress *Sirt1* transcription, silencing of HP1γ, but not HP1α or HP1β, with siRNA led to partial restoration of *Sirt1* expression in NRVMs exposed to either AA+2-DG or H_2_O_2_ (Supplementary Fig. 19). HP1γ silencing also led to the normalization of anti-oxidant genes accompanying erasure of trimethyl H3K9 and restoration of acetyl H3K9 on the *Sirt1* promoter in both AA+2-DG (Supplementary Fig. 20) and H_2_O_2_-treated (Supplementary Fig. 21) NRVMs.

**Figure 7:**
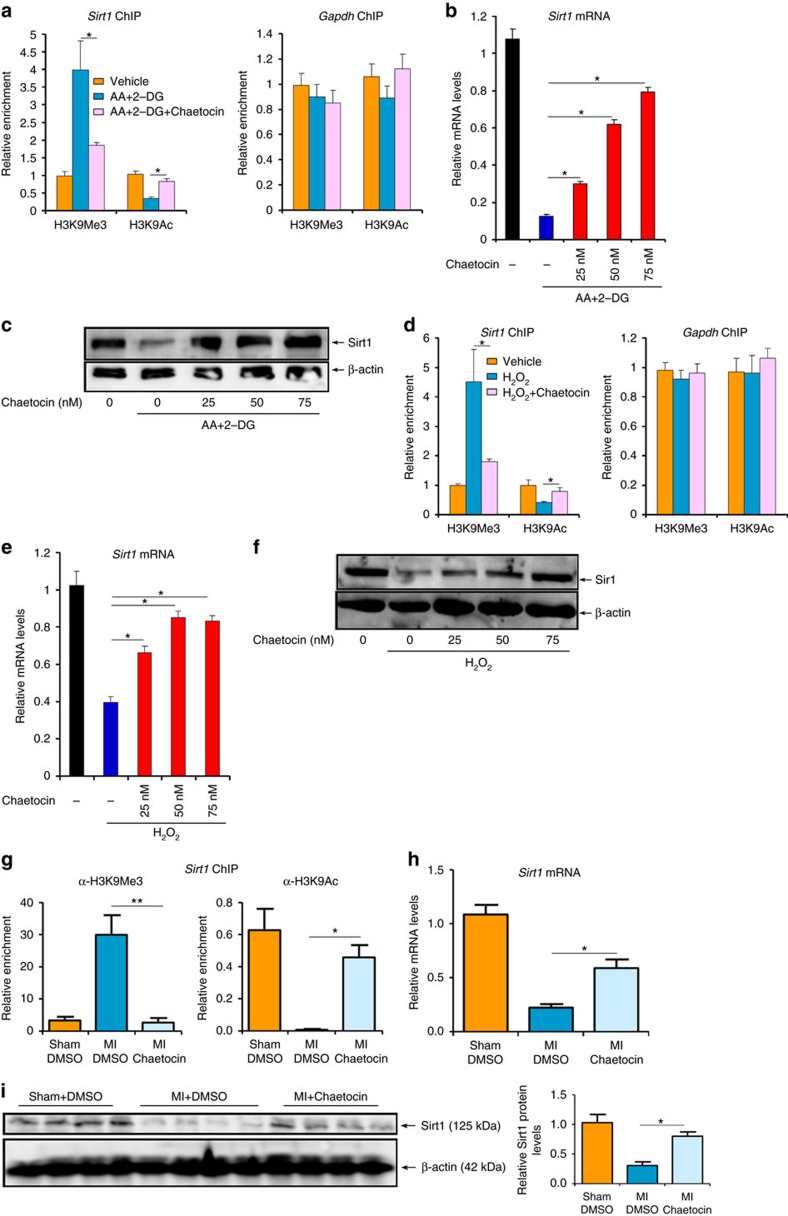
SUV39H inhibition normalizes SIRT1 transcription. (**a**–**c**) Primary NRVMs were treated with AA+2-DG or chaetocin. ChIP assays were performed with indicated antibodies (**a**). SIRT1 expression was examined by qPCR (**b**) and western blotting (**c**). (**d**–**f**) Primary NRVMs were treated with H_2_O_2_ or chaetocin. ChIP assays were performed with indicated antibodies (**d**). SIRT1 expression was examined by qPCR (**e**) and western blotting (**f**). (**g**–**i**) C57/BL mice were injected peritoneally with chaetocin (25 mg kg^−1^) or DMSO 2 days prior to the LAD procedure. ChIP assays were performed with indicated antibodies using heart homogenates (**g**). SIRT1 expression was examined by qPCR (**h**) and western blotting (**i**). Error bars represent s.d. (*N*=3 for each group). *P<0.05; **P<0.01 (one-way ANOVA with *post hoc* Scheffe test).

We then used chaetocin to inhibit SUV39H activity to validate the role of SUV39H in SIRT1 trans-repression. Indeed, chaetocin treatment simultaneously blocked H3K9Me3 accumulation and H3K9Ac erasure on the *Sirt1* promoter and dose-dependently upregulated SIRT1 in cardiomyocytes challenged with AA+2-DG (Fig. 7a–c, Supplementary Fig. 22) or H_2_O_2_ (Fig. 7d–f, Supplementary Fig. 23). Chaetocin injection in C57/BL6 mice resulted in a more active chromatin structure surrounding the *Sirt1* promoter (Fig. 7g) and increased SIRT1 expression in infarcted hearts (Fig. 7h,i).

### SUV39H regulates ROS generation in a SIRT1-dependent manner

**Figure 8:**
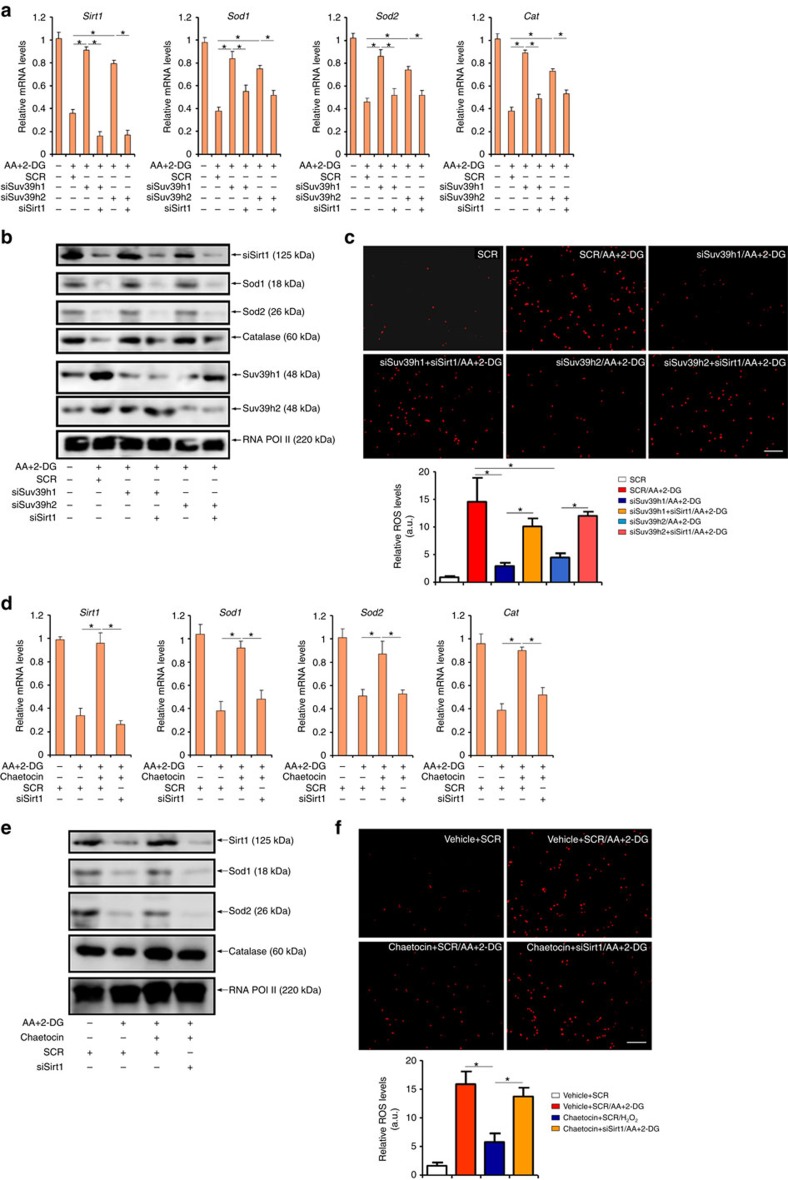
SUV39H regulates ROS generation in a SIRT1-dependent manner. (**a**–**c**) Primary NRVMs were transfected with SUV39H siRNA, SIRT1 siRNA or random siRNA (SCR) followed by treatment with AA+2-DG. Expression of anti-oxidant genes was examined by qPCR (**a**) and western blotting (**b**). Intracellular ROS levels were examined by DHE staining (**c**). Scale bar, 20 μm. (**d**–**f**) Primary NRVMs were transfected with SIRT1 siRNA, or random siRNA (SCR) followed by treatment with AA+2-DG or chaetocin. Expression of anti-oxidant genes was examined by qPCR (**d**) and western blotting (**e**). Intracellular ROS levels were examined by DHE staining (**f**). Scale bar, 20 μm. Error bars represent s.d. (*N*=3). *P<0.05 (one-way ANOVA with *post hoc* Scheffe test).

Having established that SUV39H promotes ROS generation and SIRT1 trans-repression in the process of myocardial infarction, we asked whether the ability of SUV39H to regulate ROS levels in cardiomyocytes depends on SIRT1. As shown in Fig. 8a and Fig. 8b, depletion of SUV39H blocked the downregulation of *Sod1*, *Sod2* and *Cat* by AA+2-DG treatment, which was pre-empted by siRNA-mediated SIRT1 silencing. Consistent with gene expression levels, DHE staining showed that SUV39H silencing prevented the induction of intracellular ROS generation by AA+2-DG, which was abrogated by SIRT1 inhibition (Fig. 8c). Similar results were obtained in cardiomyocytes treated with H_2_O_2_ (Supplementary Fig. 24). A specific SIRT1 inhibitor EX-527 achieved similar effects (Supplementary Figs 25 and 26). In the second set of experiments, inhibition of SUV39H activity by chaetocin alleviated the decrease of anti-oxidant gene expression by AA+2-DG (Fig. 8d,e) and the increase in intracellular ROS levels (Fig. 8f), both of which were neutralized by SIRT1 knockdown. Again, we were able to confirm that the anti-oxidative effects endowed by chaetocin were completely lost without endogenous SIRT1 activity in H_2_O_2_-treated NRVMs (Supplementary Fig. 27). Likewise, EX-527 treatment also negated the effects of chaetocin in both AA+2-DG (Supplementary Fig. 28) and H_2_O_2_-treated (Supplementary Fig. 29) NRVMs.

**Figure 9:**
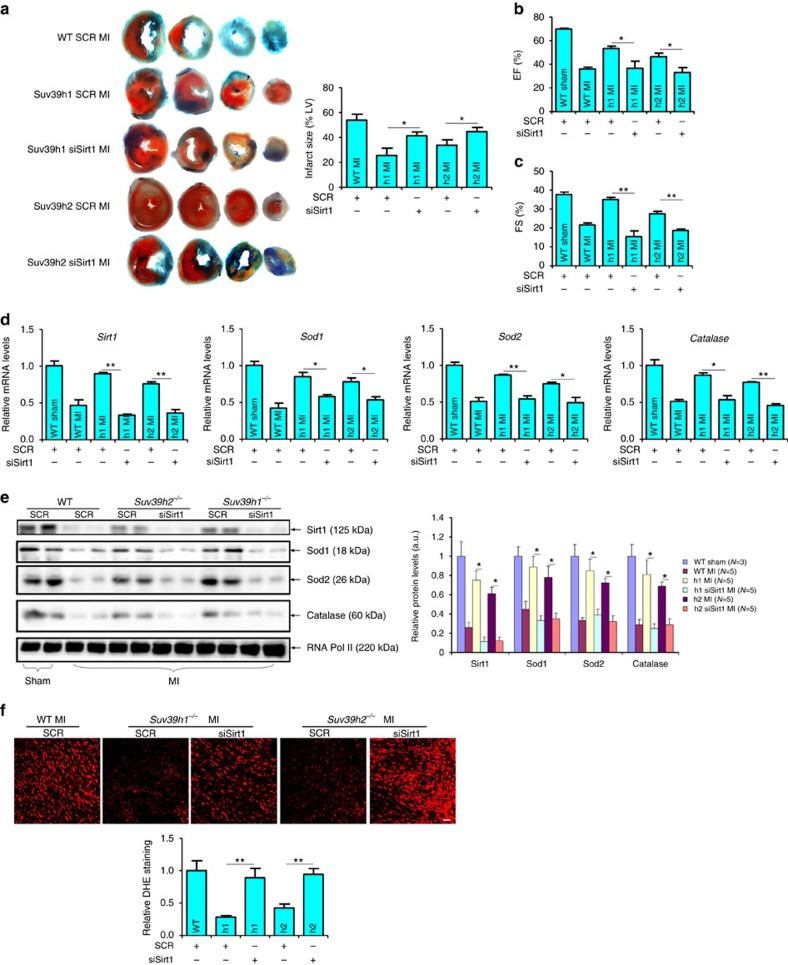
SUV39H regulates myocardial infarction by targeting SIRT1 in mice. Suv39h1 knockout (*h1*^−/−^) mice, Suv39h2 knockout (*h2*^−/−^) mice or wide-type (WT) littermates were injected via tail vein siRNA targeting SIRT1 or scrambled siRNA (SCR) followed by the LAD procedure to induce MI. (**a**) Representative TTC staining. Infarct size was calculated and quantified by Image Pro. Error bars represent s.d. (*N*=5 for each group). (**b**,**c**) EF and FS values were measured by echocardiography. Error bars represent s.d. (*N*=3 for the sham group and *N*=5 for each of the MI groups). (**d**,**e**) Expression levels of anti-oxidant genes were measured by qPCR (**d**) and western blotting (**e**). Error bars represent s.d. (*N*=3 for the sham group and *N*=5 for each of the MI groups). (**f**) Cardiac ROS levels were evaluated by DHE staining. Scale bar, 50 μm.*P<0.05; **P<0.01 (one-way ANOVA with *post hoc* Scheffe test).

**Figure 10:**
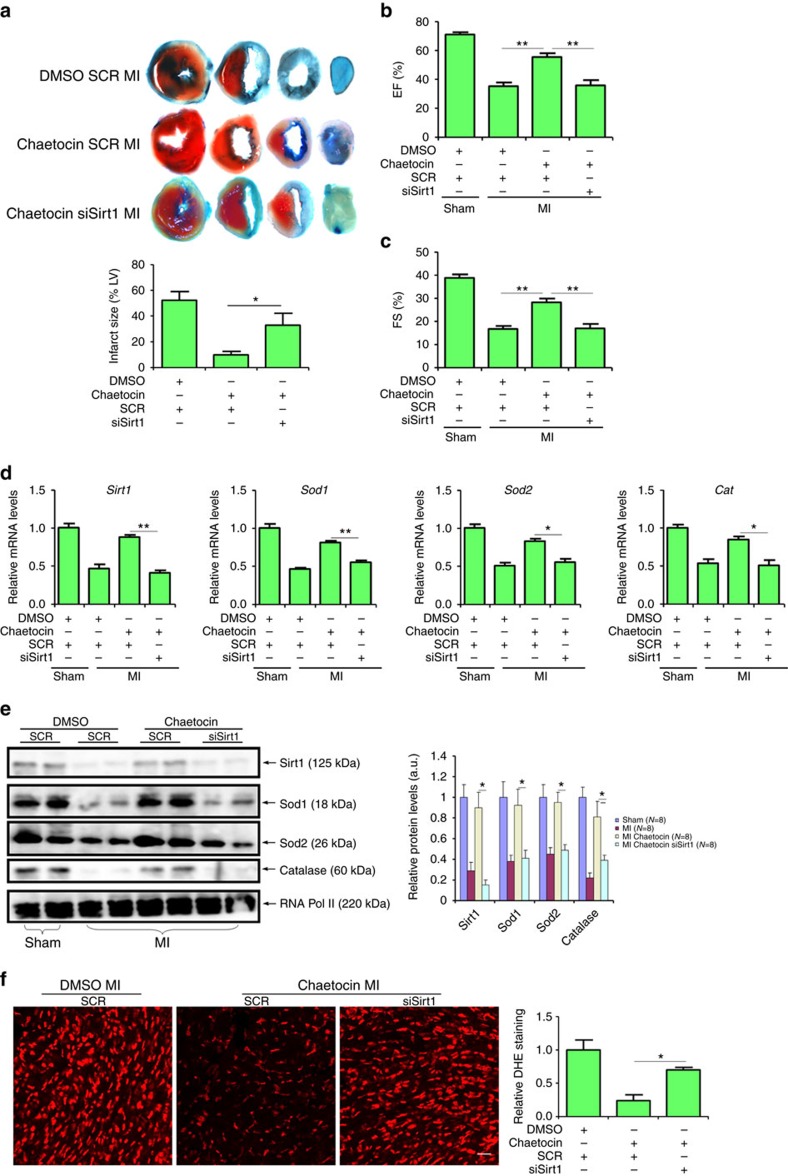
Cardioprotective effects of chaetocin depends on SIRT1 in mice. C57/BL mice were injected peritoneally with chaetocin (25 mg kg^−1^) or DMSO 2 days prior to the LAD procedure. At the same time, these mice were injected via tail vein siRNA targeting SIRT1 or scrambled siRNA (SCR). (**a**) Representative TTC staining. Infarct size was calculated and quantified by Image Pro. (**b**,**c**) EF and FS values were measured by echocardiography. (**d**,**e**) Expression levels of anti-oxidant genes were measured by qPCR (**d**) and western blotting (**e**). (**f**) Cardiac ROS levels were evaluated by DHE staining. Scale bar, 50 μm. Error bars represent s.d. (*N*=8 for each group). *P<0.05; **P<0.01(one-way ANOVA with *post hoc* Scheffe test).

Finally, we analysed whether this SUV39H–SIRT1 axis might be functional in mice. To this end, SUV39H knockout mice or wild-type littermates were injected via tail vein siRNA targeting SIRT1 or scrambled siRNA prior to the MI procedure. SIRT1 silencing almost completely blanketed the reduction in infarction size (Fig. 9a) and the improvement of post-MI heart function (Fig. 9b,c) as a result of SUV39H deficiency. qPCR and western blotting analyses indicated that in the absence of SIRT1, anti-oxidant genes were significantly downregulated (Fig. 9d,e). In addition, *Sirt1* depletion re-instated the intracellular ROS levels to those observed in wild-type hearts (Fig. 9f). Similarly, we found that the post-MI benefits associated with SUV39H deficiency in mice were largely gone by EX-6527 injection (Supplementary Fig. 30). Of note, the cardioprotective effects of chaetocin was also dependent on SIRT1 since *Sirt1* depletion (Fig. 10) or inhibition (Supplementary Fig. 31) abrogated the reduction of infarction size, the normalization of heart function, the changes in anti-oxidant gene expression, and the suppression of ROS levels induced by chaetocin in post-MI mice. Collectively, these data demonstrate that SUV39H regulates ROS generation in a SIRT1-dependent manner.

## Discussion

During MI pathogenesis, a host of insults collectively inflict damages on cardiomyocytes contributing to the loss of heart function, acute cardiac arrest and death. We present evidence that the histone H3K9 trimethyltransferase SUV39H links SIRT1 trans-repression to myocardial infarction. Using H3K9 trimethylation as a readout, Greiner *et al*.^17^ have discovered chaetocin as a specific SUV39H inhibitor. Chaetocin has since been shown to confer protection against hepatoma^18^ and leukaemia^19^ in mice although the mechanisms seem to be multifold. It remains ambiguous whether the effects of chaetocin are entirely mediated through SUV39H and the specificity of chaetocin has been called into question^20^. Similar to our study, Schweizer *et al*.^21^ have reported that chaetocin administration is associated with neuroprotection in an *in vitro* model of cerebral ischaemia. In addition, these authors demonstrated that chaetocin treatment activates the transcription of neurotrophin genes (BDNF) at least in part by stimulating H3K9 acetylation across the BDNF promoters. These data echo our own observation that chaetocin protects ischaemia-challenged cardiomyocytes by epigenetically activating *Sirt1* transcription (Fig. 7) and suggest that at least in the context of ischaemia-induced cell damages, chaetocin may be equivalent to SUV39H silencing/depletion.

More recently, Cotman and colleagues have identified SUV39H-mediated H3K9 trimethylation as a critical link between ageing and memory loss in mice^22^. Of importance, this group of investigators exploited a more selective SUV39H inhibitor called ETP69 to show that SUV39H inhibition significantly improved learning of complex task in aged mice. Not coincidently, MI occurs predominantly in the aged population. In the light of our finding that chaetocin injection ameliorates the loss of heart function following MI in mice (Fig. 4), these observations collectively allude to a scenario wherein SUV39H modulates ageing-related pathophysiological processes by epigenetically regulating key transcription events. Indeed, a report by Liu *et al*.^23^ indicates that SUV39H1 deficiency circumvents premature ageing in *Zmpste24*^−/−^ mice that model the Hutchinson–Gilford progeria syndrome in humans, offering strong support for the argument that SUV39H activity promotes ageing and ageing-associated organ dysfunctions such as dementia and MI.

Curiously, Schweizer *et al*. and Liu *et al*. only examined systemic levels of H3K9Me3, which from a transcriptional perspective bears little, if any, relevance to the actual molecular proceedings underlying disease pathogenesis. A couple of investigations profiling genome-wide patterns of H3K9Me3 enrichment have offered conflict but insightful snapshots at the role of SUV39H in different pathobiological processes. Using hippocampus from acutely stressed Sprague-Dawley rats, Hunter *et al*. showed while the transposable and repeated regions saw a significant increase in H3K9Me3 binding upon stress, little change was found for H3K9Me3 in the gene body (for example, promoter regions)^24^, suggesting that SUV39H most likely plays a very limited role regulating transcription in the euchromatin region. In contrast, a group led by Casaccia has reported that during oligodendrocyte differentiation, a vast majority of H3K9Me3 can be mapped to the gene body encoding proteins involved in neuronogenesis^25^. We show here that SUV39H expression/activity is intimately connected to *Sirt1* promoter-specific H3K9Me3 levels. In addition, we show that HP1γ, but not HP1α or HP1β, co-regulates SIRT1 trans-repression with SUV39H. Unlike HP1α or HP1β, HP1γ is exclusively situated in the euchromatin region^26^. These data suggest that SUV39H may regulate euchromatin transcription in a tissue- and context-specific manner likely guided by HP1γ. ChIP-seq analyses performed in healthy and infarcted myocardium may help clarify this issue and assign a more precise role for SUV39H in MI pathogenesis.

An intriguing observation here is that *Suv39h1* deficiency seems to confer bigger advantage to mice in terms of survival, infarct size, post-MI heart function and ROS levels than *Suv39h2* deficiency (Fig. 3), all of which could probably be attributed to the fact that SUV39H1 acts as a more potent repressor of *Sirt1* transcription (Figs 5 and 6). In response to MI-relevant stress stimuli, the expression of SUV39H1 and SUV39H2 was altered with distinctive kinetics: SUV39H1 upregulation was more persistent while SUV39H2 upregulation was more transient (Figs 1 and 2). In addition, HP1γ appears to preferentially interact with SUV39H1 on the *Sirt1* promoter (Supplementary Fig. 10), which may also explain why SUV39H1 played a more dominant role in SIRT1 trans-repression. It has been previously shown that SUV39H1 and SUV39H2 redundantly maintain global H3K9Me3 levels, which are completely erased in SUV39H1/H2 double knockout (DN) mice^27^. It remains to be elucidated how SUV39H1 and SUV39H2 differentially bind to target genes to regulate MI pathogenesis. SUV39H2 only shares ∼60% amino-acid sequence identity with SUV39H1 with a highly variable N-terminal domain and the structural differences may determine target promoter selection by SUV39H1 and SUV39H2 (ref. 28). Alternatively, SUV39H1 and SUV39H2 may have non-overlapping lists of binding partners, which in turn may steer them to different chromatin regions to regulate transcription, because of distinct structures. A recent report by the Jenuwein group provides some support for this model: SUV39H-depednent heterochromatin formation relies on the sequence-specific transcription factors PAX3 and PAX9 that bind preferentially to the intergenic satellite repeats^29^. Since the PAX family of proteins including PAX3 and PAX9 possess well-defined functions in regulating a range of pathophysiological processes in the cardiovascular system^30^, we suspect a similar scheme may exist in the euchromatin region to control SUV39H target selection.

At the cellular level, we focused on the role of SUV39H in regulating ROS levels in cardiomyocytes, which is unlikely to reflect in full the realm that SUV39H reigns with regards to MI pathogenesis. For instance, lymphocyte mediated immune response plays a pivotal role in regulating MI pathogenesis encompassing injury, healing and remodelling^31^. By directly regulating the transcription of lineage specification genes, SUV39H1 has been show to strike a balance between different T-lymphocyte subsets^32^. In addition, SUV39H1 has also been shown to impact B-lymphocyte-dependent humoral immune response by modulating class switch recombination^33^. These observations highlight a potential role for SUV39H in shaping up the immune response during MI pathogenesis. Furthermore, a string of recent reports have put SUV39H at center stage in regulating stem cell phenotype^34^,^35^,^36^, an increasingly promising target for improving post-MI heart function. Of interest, bone marrow transplantation experiments (Supplementary Figs 7 and 8) indicate that myeloid-derived SUV39H is unlikely to play a major role in MI pathogenesis. SUV39H has been reported to repress the transcription of p21 (ref. 37) and PML-RAR^38^ target genes in macrophages although relatively little is known regarding the systemic regulatory role of SUV39H in myeloid cells in the context of MI. Further confounding the link between SUV39H and SIRT1 is the inherent weakness of some of our methodologies. In addition to the systemic, instead of lineage-specific, knockout models, we have used tail vein injection to deliver siRNAs, which may lodge in tissues and organs other than the heart such that a definitive relationship between SUV39H and SIRT1 cannot be taken for granted. Clearly, the cell-specific roles of SUV39H in MI pathogenesis should be revisited taking into account of the aforementioned possibilities when conditional knockout animals are made available.

Previous investigations have examined the functional relationship between SUV39H1 and SIRT1 in different contexts. For instance, Murayama *et al*.^39^ have reported that SIRT1 forms a co-repressor complex with SUV39H1 to repress the transcription of rRNA in response to intracellular energy status. SIRT1 has also been found to cooperate with SUV39H1 to modulate the chromatin structure surrounding the leukaemic genes and prevent MLL-rearranged leukaemia^40^. Vaquero *et al*. have shown that SIRT1 could directly modify (deacetylate) and thus stabilize SUV39H1 to maintain the integrity of heterochromatin^41^,^42^. Our finding adds an additional layer to the intricate SIRT1–SUV39H1 interplay. It would be of key interest to see how these different modes of actions dynamically contribute to the regulation of ischaemic heart disease.

In summary, our data demonstrate the potential of SUV39H deletion/inhibition in alleviating damages associated with MI. A genomewide search for more SUV39H targets, using both RNA-seq and ChIP-seq techniques, in different types of cells in the context of MI would hopefully clear the lingering uncertainties and render a more rationalized decision to target SUV39H for drug development.

## Methods

### Cell culture and treatment

Immortalized rat myoblast cells (H9C2, ATCC CRL-1446) were maintained in DMEM (Invitrogen) supplemented with 10% fetal bovine serum (FBS, Hyclone). Primary rat NRVMs were isolated from 1-day-old Sprague-Dawley rats by digestion with type 2 collagenase. Cells were cultured in serum-free insulin-transferrin (IT) medium (21) for an additional 24–36 h (ref. 43). Antimycin (AA), 2-deoxy glucose (2-DG), and hydrogen peroxide (H_2_O_2_) and chaetocin were purchased from Sigma.

### Plasmids and transient transfection

Myc-tagged SUV39H1 expression constructs^41^, HA-tagged HP1 expression constructs^44^ and SIRT1 promoter-luciferase construct^45^ have been previously described. Small interfering RNA (siRNA) sequences were as follows: for Suv39h1, 5′-ccacggcagaaucuaaautt-3′; for Suv39h2, 5′-ggaccugauugucccaauatt-3′; for Hp1a, 5′-GCUUUGAGAGAGGACUGGAACTT-3′; for Hp1b, 5′-GACUCCAGUGGAGAGCUCAUGTT-3′; for Hp1g, 5′-GAGGCAGAGCCUGAAGAAUTT-3′ and for Sirt1, 5′-CACCUGAGUUGGAUGAUAUTT-3′. Transient transfections were performed with Lipofectamine 2000 (Invitrogen). Luciferase activities were assayed 24–48 h after transfection using a luciferase reporter assay system (Promega). Experiments were routinely performed in triplicate wells and repeated three times.

### Mice

Suv39h1 knockout and Suv39h2 knockout mice have been previously described^27^. All protocols were approved by the intramural Committee on Ethic Conduct of Animal Studies of Nanjing Medical University. MI was induced by permanent ligation of the LAD coronary artery in male, 8-week-old mice. Briefly, the mice were anaesthetized with a mixture of ketamine (120 mg kg^−1^) and xylazine (6 mg kg^−1^), the hearts were exposed and the LAD was ligated with a 6-0 prolene suture. The control mice were sham operated wherein the ligature around the LAD was not tied. Post-MI heart functions were evaluated by echocardiography (GE Vivid 7 equipped with a 14-MHz phase array linear transducer, S12, allowing a 150 maximal sweep rate). Myocardial infarct size was determined by Evans blue/triphenyltetrazolium chloride (TTC) staining^46^. Briefly, the hearts were perfused with saline on a Langendorff system to remove blood and then stained with with 1% Evans blue followed by incubation in 1% TTC-Tris solution) for 15 min at 37 °C. For ROS measurements, frozen heart sections (4 μm) were stained with DHE (2 μmol l^−1^, Sigma) in a light-protected humidified chamber at 37 °C for 15 min. The slides were visualized by co-focal fluorescence microscopy (Zeiss). To examine cardiomyocyte apoptosis, frozen heart sections (4-μm) were stained using a TUNEL kit (Roche) and the nuclei were counter-stained with DAPI (Sigma). The slides were visualized by co-focal fluorescence microscopy (Zeiss).

### Protein extraction and western blot

Whole-cell lysates were obtained by re-suspending cell pellets in RIPA buffer (50 mM Tris pH 7.4, 150 mM NaCl, 1% Triton X-100) with freshly added protease inhibitor cocktail tablet (Roche). Western blot analyses were performed with anti-Suv39h1 (Proteintech, 10574-1, 1:1,000), anti-Suv39h2 (Genetex, GTX129167, 1:1,000), anti-HP1α (Proteintech, 11831-1, 1:1,000), anti-HP1β (Proteintech, 10241-1, 1:1,000), anti-HP1γ (Proteintech, 11650-2, 1:1,000), anti-SOD1 (Proteintech, 10269-1, 1:1,000), anti-SOD2 (Proteintech, 24127-1, 1:1,000), anti-catalase (Proteintech, 21260-1, 1:1,000), anti-α-SMA (Sigma, F3777, 1:5,000), anti-SIRT1 (Abcam, ab110304, 1:2,000), anti-collagen type I (Rockland, 001-001-103, 1:2,000), RNA Polymerase II (Santa Cruz, SC-899, 1:2,000) and anti-β-actin (Sigma, A5441, 1:5,000) antibodies. Uncropped western blots are presented in Supplementary Fig. 32.

### RNA isolation and real-time PCR

RNA was extracted with the RNeasy RNA isolation kit (Qiagen). Reverse transcriptase reactions were performed using a SuperScript First-strand Synthesis System (Invitrogen). Real-time PCR reactions were performed on an ABI Prism 7500 system. Primers and Taqman probes used for real-time reactions were purchased from Applied Biosystems.

### ChIP and Re-ChIP

ChIP and Re-ChIP assays were performed as previously described^47^,^48^,^49^. Aliquots of lysates containing 200 μg of protein were used for each immunoprecipitation reaction with anti-SUV39H1 (Abcam, ab12405, 5 μg per reaction), anti-SUV39H2 (Abcam, ab5264, 5 μg per reaction), anti-trimethylated H3K9 (Abcam, ab8898, 2 μg per reaction), anti-acetyl H3K9 (Abcam, ab10812, 2 μg per reaction), anti-HP1α (Abcam, ab10811, 5 μg per reaction), anti-HP1β (Abcam, ab77256, 5 μg per reaction) and anti-HP1γ (Abcam, ab154871, 5 μg per reaction). For re-ChIP, immune complexes were eluted with the elution buffer (1% SDS, 100 mM NaHCO_3_), diluted with the re-ChIP buffer (1% Triton X-100, 2 mM EDTA, 150 mM NaCl, 20 mM Tris pH 8.1), and subject to immunoprecipitation with a second antibody of interest. Precipitated genomic DNA was amplified by real-time PCR with the following primers: *Sirt1* promoter region (−182/+56), 5′-GCCATCGCAAACTTGAACCACC-3′ and 5′-CGTCCGCCATCTTCCAACTGC-3′; Gapdh promoter region (−225/+31), 5′-ATCACTGCCACCCAGAAGACTGTGGA-3′ and 5′-CTCATACCAGGAAATGAGCTTGACAAA-3′; *Sod1* promoter region (−212/+18), 5′-AATAGCGACTTTCCCAGCTC-3′ and 5′-AAACGAAGGTGCAAAACGAG-3′.

### Statistical analysis

Animal numbers and sample sizes reflected the minimal number needed for statistical significance based on power analysis and prior experience. No data were excluded from any of the experiments. Grouping was performed in a randomized manner. Blinding were not performed because it was not appropriate for the types of animal groups used here, or the types of comparisons used between groups. One-way analysis of variance with *post hoc* Scheffe analyses were performed using an SPSS package. Mouse survival data was analysed by the Kaplan–Meier log-rank test.

### Data availability

All data generated or analysed during this study are included in this published article and its Supplementary Information files, or are available from the corresponding author on reasonable request.

## Additional information

**How to cite this article:** Yang, G. *et al*. The histone H3K9 methyltransferase SUV39H links SIRT1 repression to myocardial infarction. *Nat. Commun.* **8,** 14941 doi: 10.1038/ncomms14941 (2017).

**Publisher's note:** Springer Nature remains neutral with regard to jurisdictional claims in published maps and institutional affiliations.

## Supplementary Material

Supplementary Information Supplementary Figures and Supplementary Table.
