# Supplementary material for: The histone H3K9 methyltransferase SUV39H links SIRT1 repression to myocardial infarction
Source: Nat Commun. 2017 Mar 31;8:14941. doi: 10.1038/ncomms14941 (PMC5381011; doi:10.1038/ncomms14941)
Supplement: Supplementary Information — Supplementary Figures and Supplementary Table. [file ncomms14941-s1.pdf]

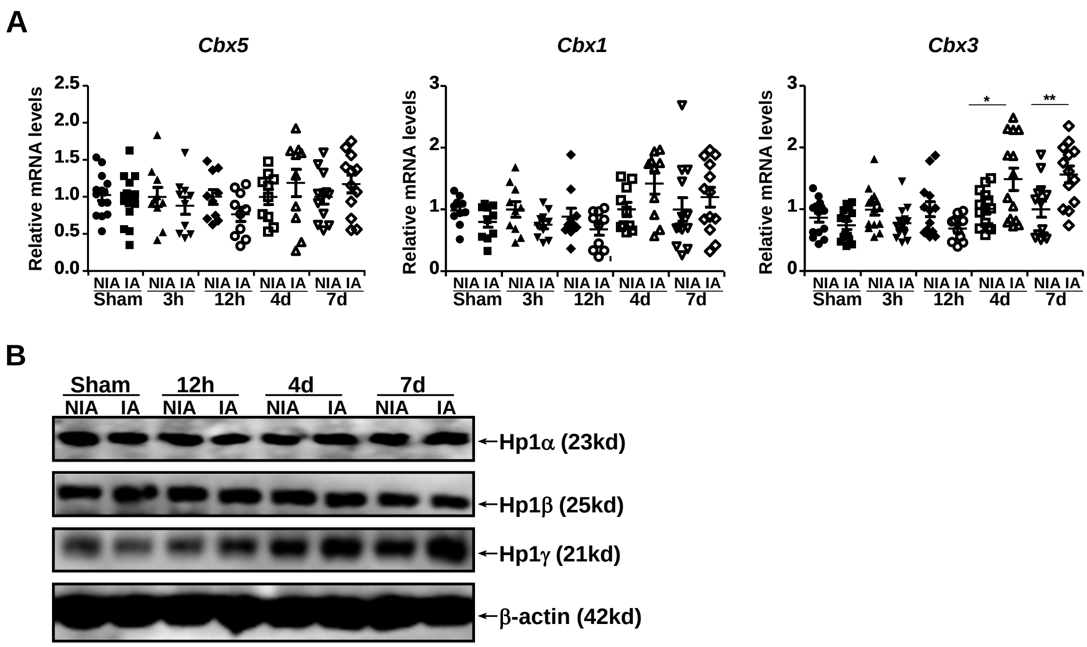

Supplementary Fig.1: ***HP1γ levels were up-regulated in the heart following MI.*** MI was induced in C57/BL6 mice as described in Methods. Expression levels of HP1g in infarct area (IA) and non-infarct area (NIA) were examined by qPCR (A) and Western (B). Error bars represent standard deviation (N=10 mice for each group). *, p＜.05; **, p＜.01 (One-way ANOVA with post-hoc Scheffe test).

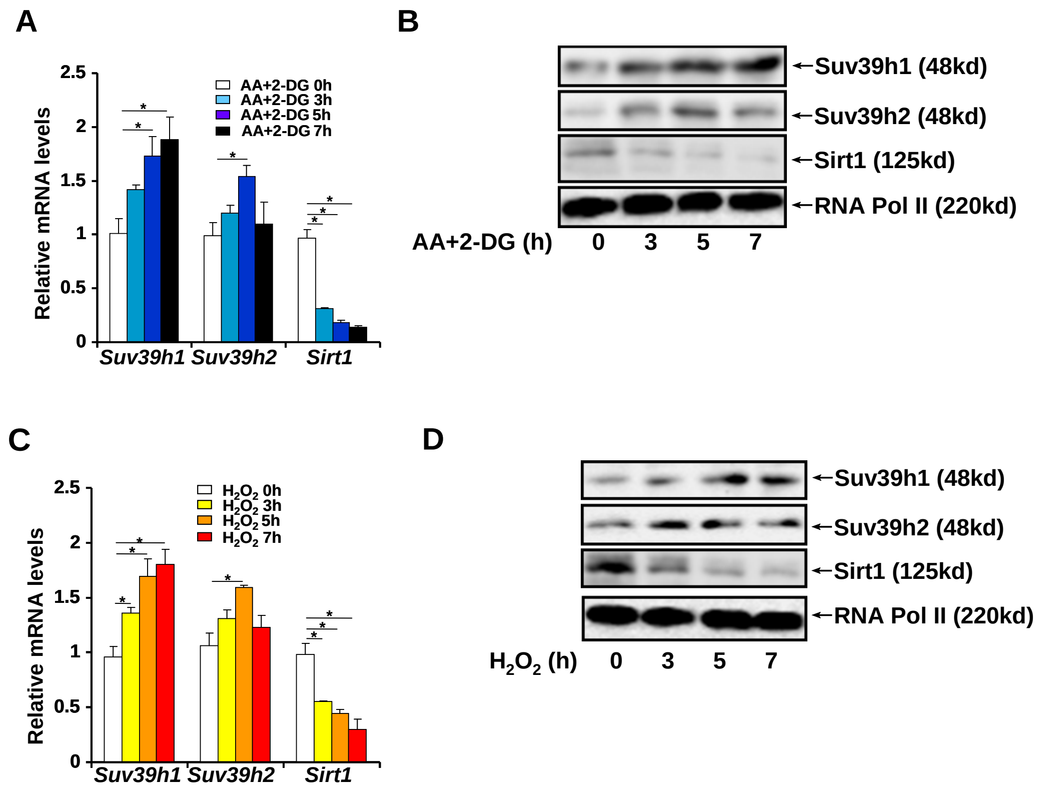

Supplementary Fig.2: ***SUV39H levels were up-regulated by ischemic and oxidative stimuli in H9C2 cells***. (**A, B**) H9C2 cells were treated with AA+2-DG and harvested at indicated time points. Expression levels were examined by qPCR (A) and Western (B). (**C, D**) H9C2 cells were treated with H_2O_2 and harvested at indicated time points. Expression levels were examined by qPCR (C) and Western (D). Error bars represent standard deviation (N=3). *, p＜.05; **, p＜.01 (One-way ANOVA with post-hoc Scheffe test).

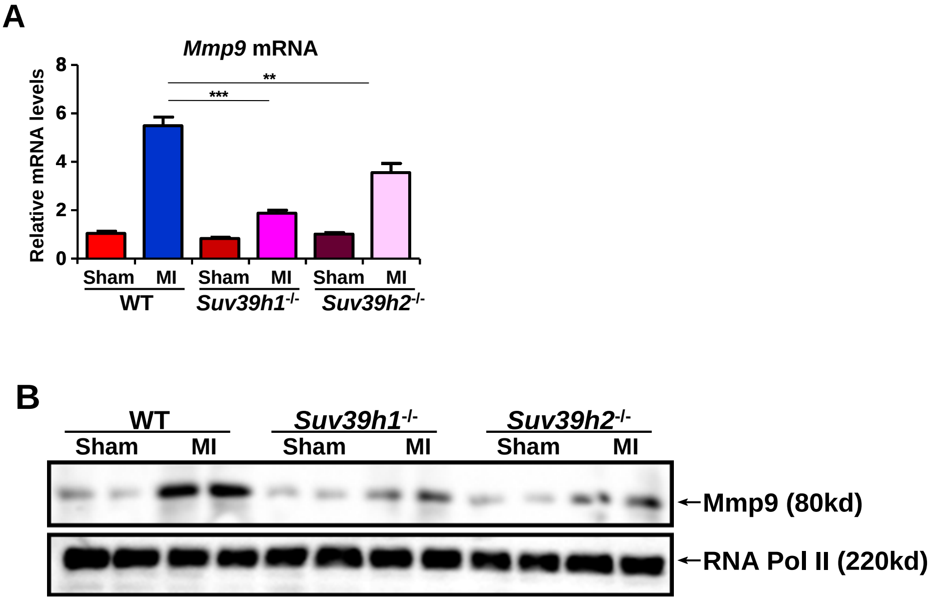

Supplementary Fig.3: (**A, B**) MI was induced in Suv39h1 knockout mice, Suv39h2 knockout mice, or wide type (WT) littermates by LAD. MMP9 expression was evaluated by qPCR and Western. Error bars represent standard deviation (N=4 each for the sham groups and =8 each for MI groups). *, p＜.05; **, p＜.01 (One-way ANOVA with post-hoc Scheffe test).

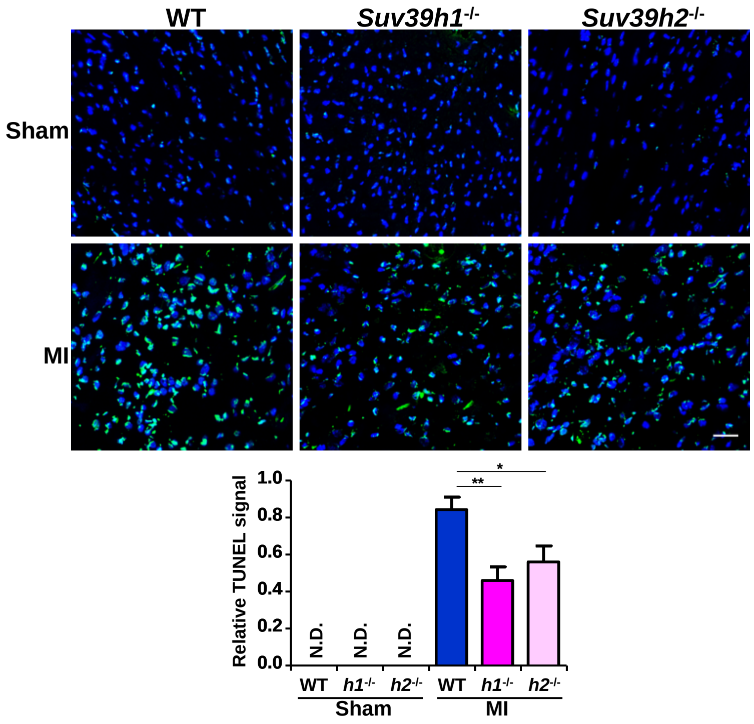

Supplementary Fig.4: MI was induced in Suv39h1 knockout mice, Suv39h2 knockout mice, or wide type (WT) littermates by LAD. TUNEL assay was performed as described in Methods and quantified by Image Pro. N.D., not detected. Scale bar, 25μm. Error bars represent standard deviation (N=3 each for the sham groups and =4 each for MI groups). *, $p < .05$; **, $p < .01$ (One-way ANOVA with post-hoc Scheffe test).

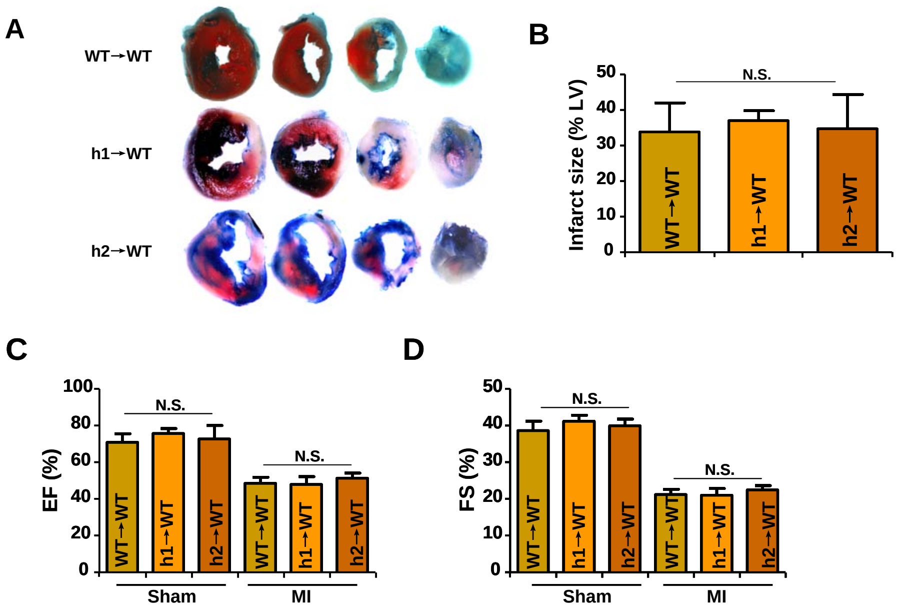

Supplementary Fig.5: ***Myeloid-derived SUV39H plays minimal role in myocardial infarction***. MI was induced following bone marrow transplantation as described in Methods. (**A**) Representative TTC staining. (**B**) Infarct size was calculated and quantified by Image Pro. (**C, D**) EF and FS values were measured by echocardiography. Error bars represent standard deviation (N=3 each for the sham groups and =5 each for the MI groups). N.S., no statistical significance (One-way ANOVA with post-hoc Scheffe test).

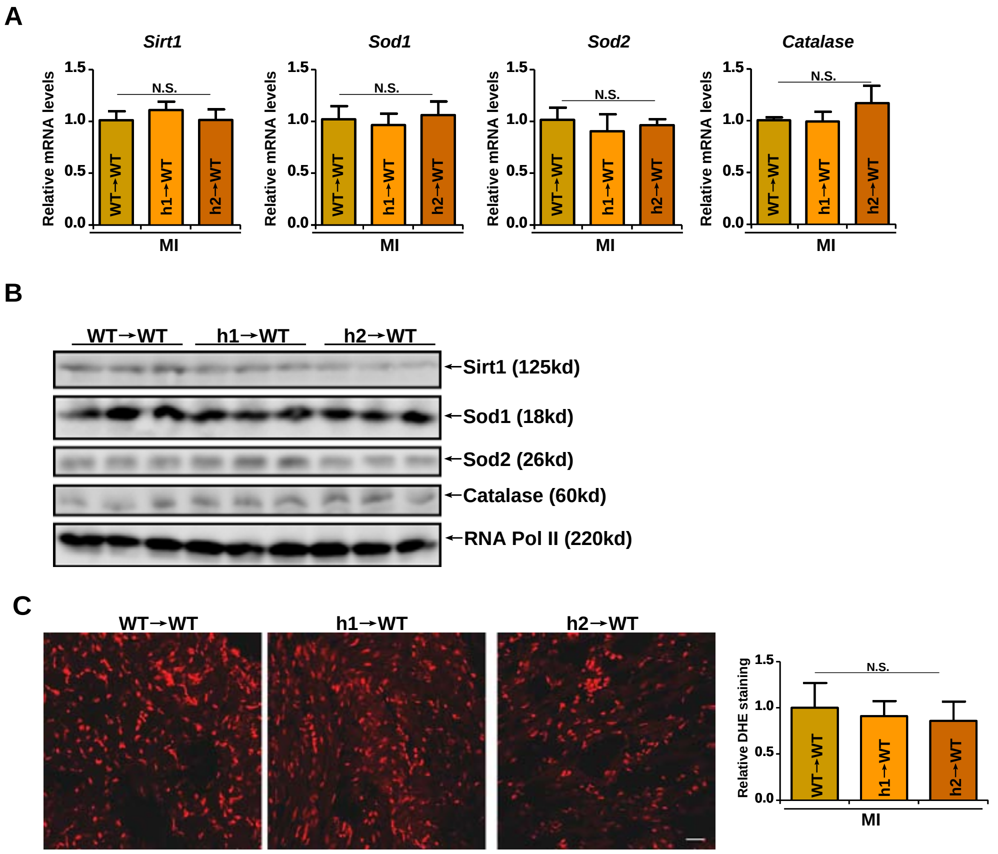

Supplementary Fig.6: ***Myeloid-derived SUV39H plays minimal role in myocardial infarction***. MI was induced following bone marrow transplantation as described in Methods. (**A, B**) Expression levels of antioxidant genes were measured by qPCR (A) and Western (B). (**C**) Cardiac ROS levels were evaluated by DHE staining. Error bars represent standard deviation (N=5 each for the MI groups). N.S., no statistical significance (One-way ANOVA with post-hoc Scheffe test).

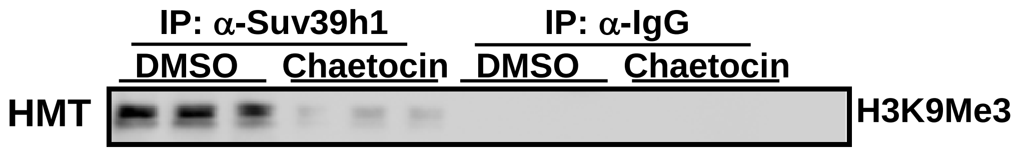

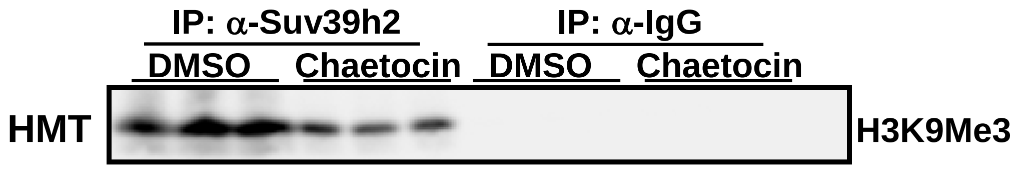

Supplementary Fig.7: ***Chaetocin injection inhibits SUV39H activity in mice*.** C57/BL mice were injected peritoneally with chaetocin (25mg/kg) or DMSO for 2 days. SUV39H was immunoprecipitated from heart homogenates with indicated antibodies; IgG was included as a negative control. HMT assays were performed as described in Methods.

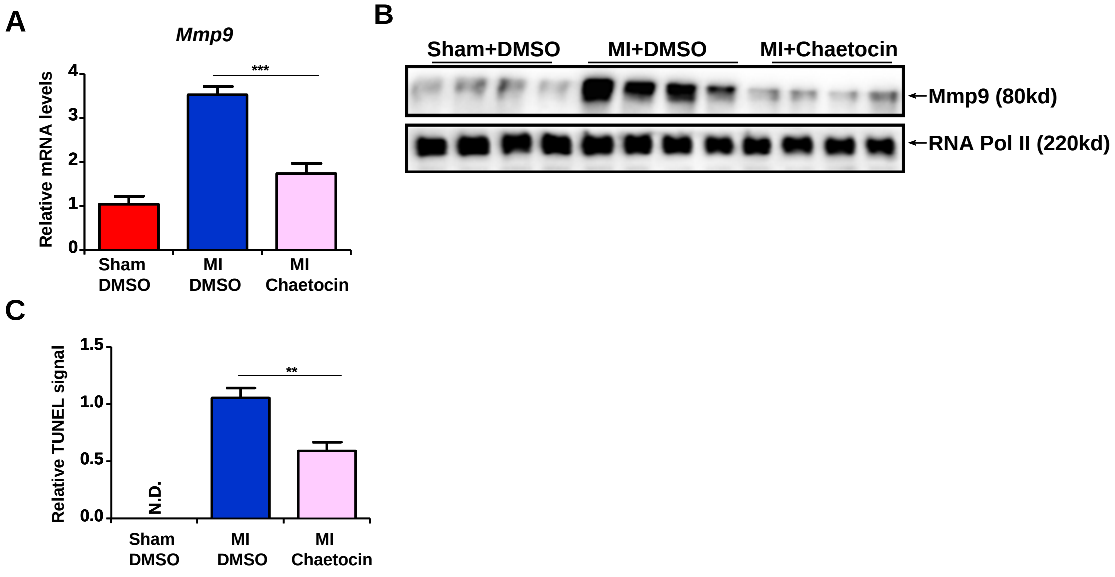

Supplementary Fig.8: C57/BL mice were injected peritoneally with chaetocin (25mg/kg) or DMSO 2 days prior to the LAD procedure. The mice were sacrificed 7 days after the surgery. (**A, B**) MMP9 expression was examined by qPCR (A) and Western (B). Error bars represent standard deviation (N=4 for each group). (**C**) TUNEL assay was performed as described in Methods and quantified by Image Pro. Error bars represent standard deviation (N=3 for each group). **, $p < .01$; ***, $p < .001$ (One-way ANOVA with post-hoc Scheffe test).

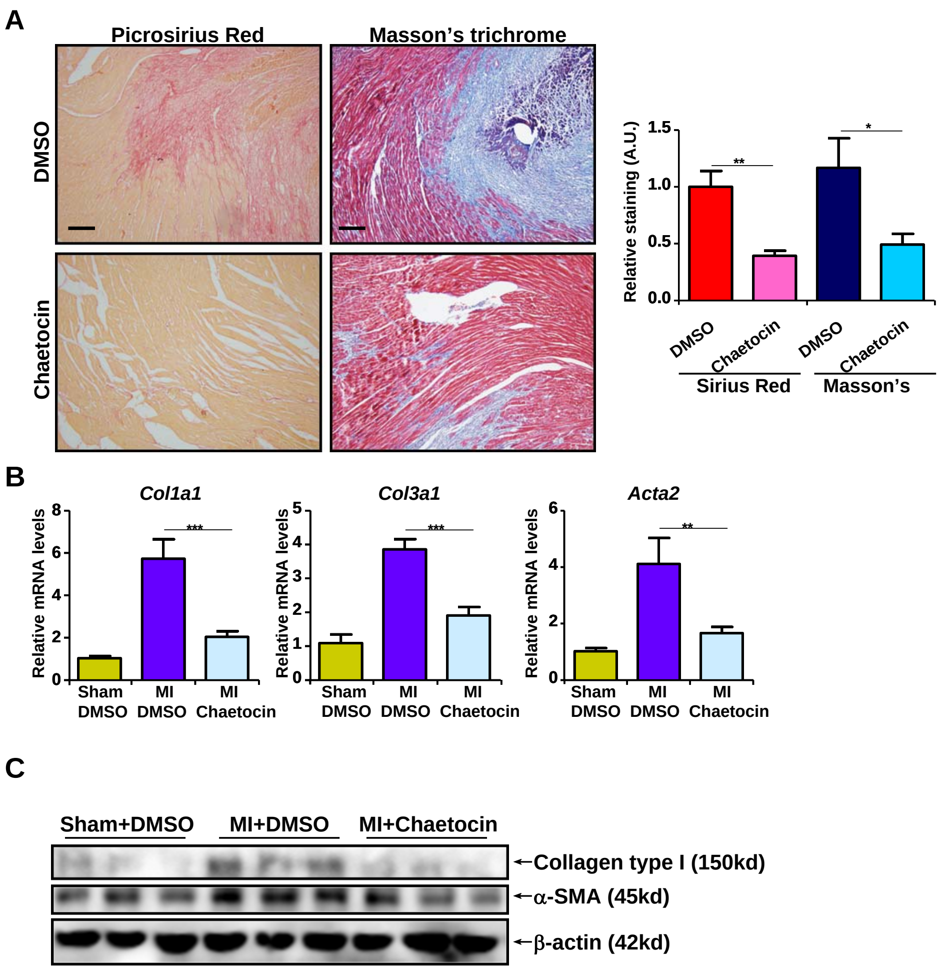

Supplementary Fig.9: C57/BL mice were injected peritoneally with chaetocin (25mg/kg) or DMSO 2 days prior to the LAD procedure. The mice were sacrificed 4 weeks after the surgery. (**A**) Paraffin-embedded heart sections were stained with picrosirius red and Masson's trichrome. Error bars represent standard deviation (N=3 for each group). (**B, C**) Expression of pro-fibrogenic genes was examined by qPCR and Western blotting. Error bars represent standard deviation (N=4 for each group). *, $p<.05$; **, $p<.01$; ***, $p<.001$ (One-way ANOVA with post-hoc Scheffe test).

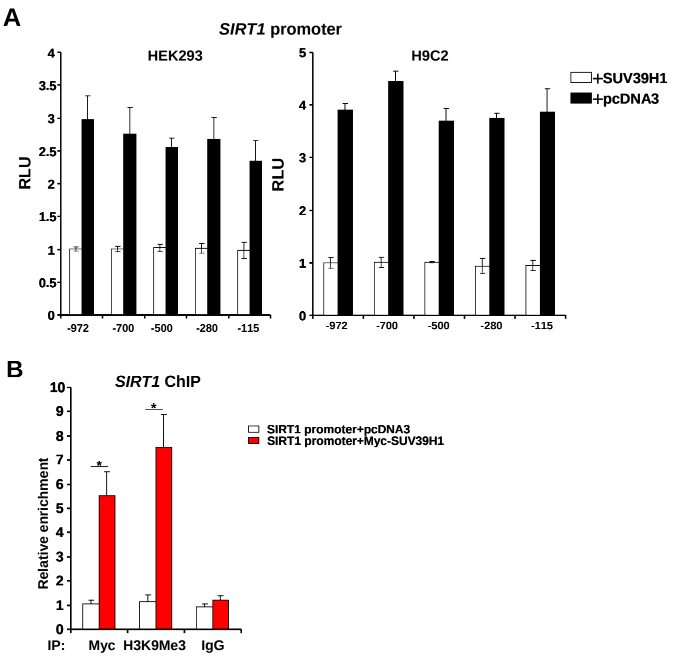

Supplementary Fig.10: ***SUV39H1 represses SIRT1 promoter activities.*** (**A**) SIRT1 promoter constructs of different lengths were transfected into HEK293 cells or H9C2 cells along with a SUV39H1 expression construct or an empty vector (pcDNA3). Luciferase activities were normalized by both protein concentration and GFP fluorescence and expressed as relative luciferase unit. (**B**) A SIRT1 promoter construct (-115) was transfected into H9C2 cells along with a SUV39H1 expression construct or an empty vector (pcDNA3). ChIP assays were performed with indicated antibodies. Error bars represent standard deviation (N=3). *, p ＜.05 (One-way ANOVA with post-hoc Scheffe test).

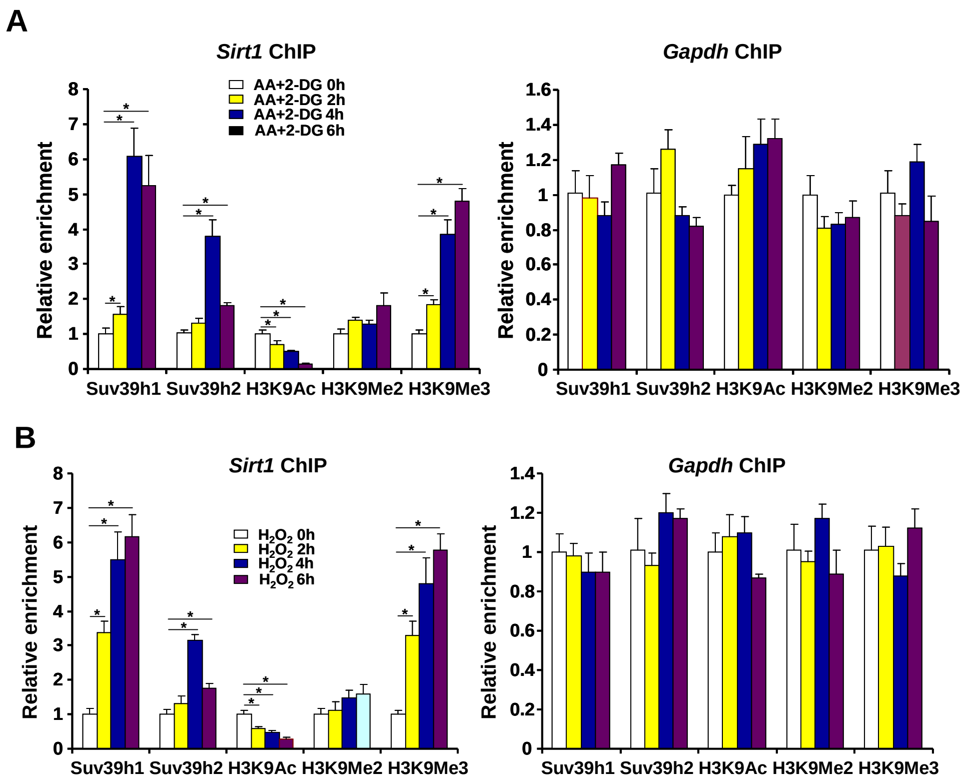

Supplementary Fig.11: ***SUV39H is recruited to the SIRT1 promoter*****.** H9C2 cells were treated with AA plus 2-DG (A) or H□O2 (B). Cells were harvested at indicated time points and ChIP assays were performed with indicated antibodies. Error bars represent standard deviation (N=4 for each group). Error bars represent standard deviation (N=3). *, p＜.05 (One-way ANOVA with post-hoc Scheffe test).

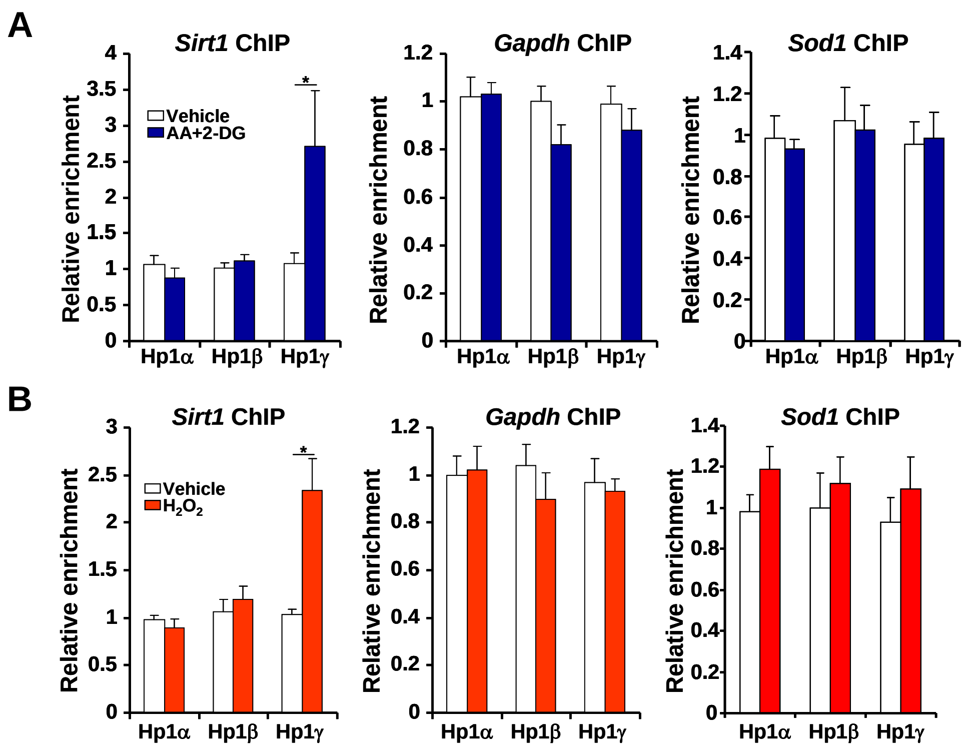

Supplementary Fig.12: ***HP1γ is specifically recruited to the SIRT1 promoter region.*** Primary NRVMs were treated with AA+2-DG (A) or H_2O_2 (B). ChIP assays were performed with indicated antibodies. Error bars represent standard deviation (N=3). *, $p < .05$ (One-way ANOVA with post-hoc Scheffe test).

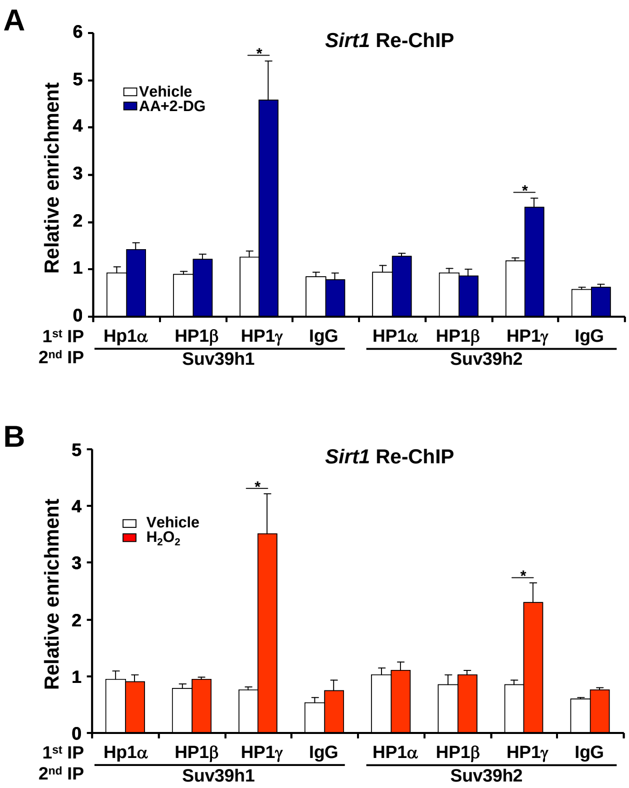

Supplementary Fig.13: ***HP1γ specifically interacts with SUV39H on the SIRT1 promoter region.*** (**A, B**) Primary NRVMs were treated with AA plus 2-DG (A) or H_2O_2 (B). Re-ChIP assays were performed with indicated antibodies. Error bars represent standard deviation (N=3). *, p＜.05 (One-way ANOVA with post-hoc Scheffe test).

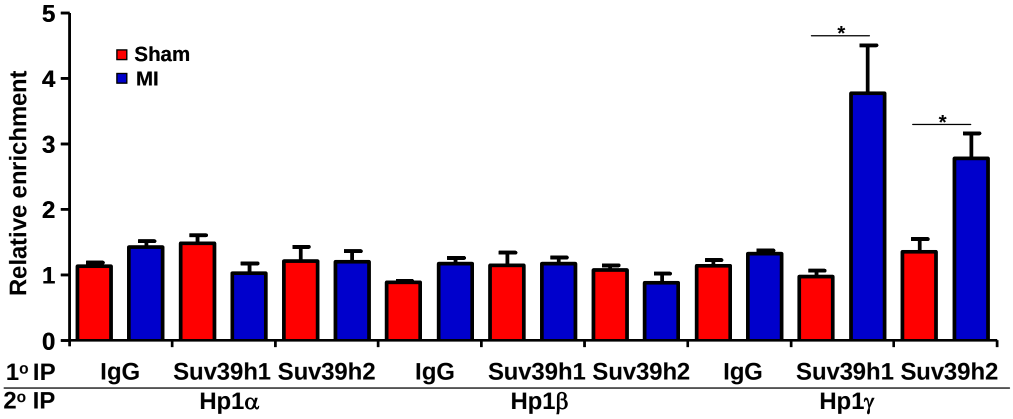

Supplementary Fig.14: ***HP1γ interacts with Suv39h1/h2 on the Sirt1 promoter in vivo***. Re-ChIP assays were performed with heart homogenates from MI mice or sham mice. Error bars represent standard deviation (N=3). *, $p < .05$ (One-way ANOVA with post-hoc Scheffe test).

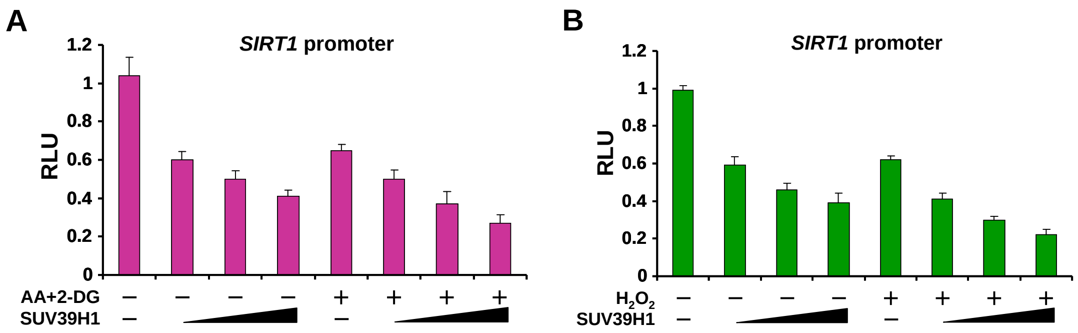

Supplementary Fig.15: ***SUV39H represses SIRT1 promoter activity.*** (**A, B**) A SIRT1 promoter construct (-115/+54) was transfected into H9C2 cells along with a SUV39H1 expression construct followed by treatment with either AA+2-DG (A) or H_2O_2 (B). Luciferase activities were normalized by both protein concentration and GFP fluorescence and expressed as relative luciferase unit.

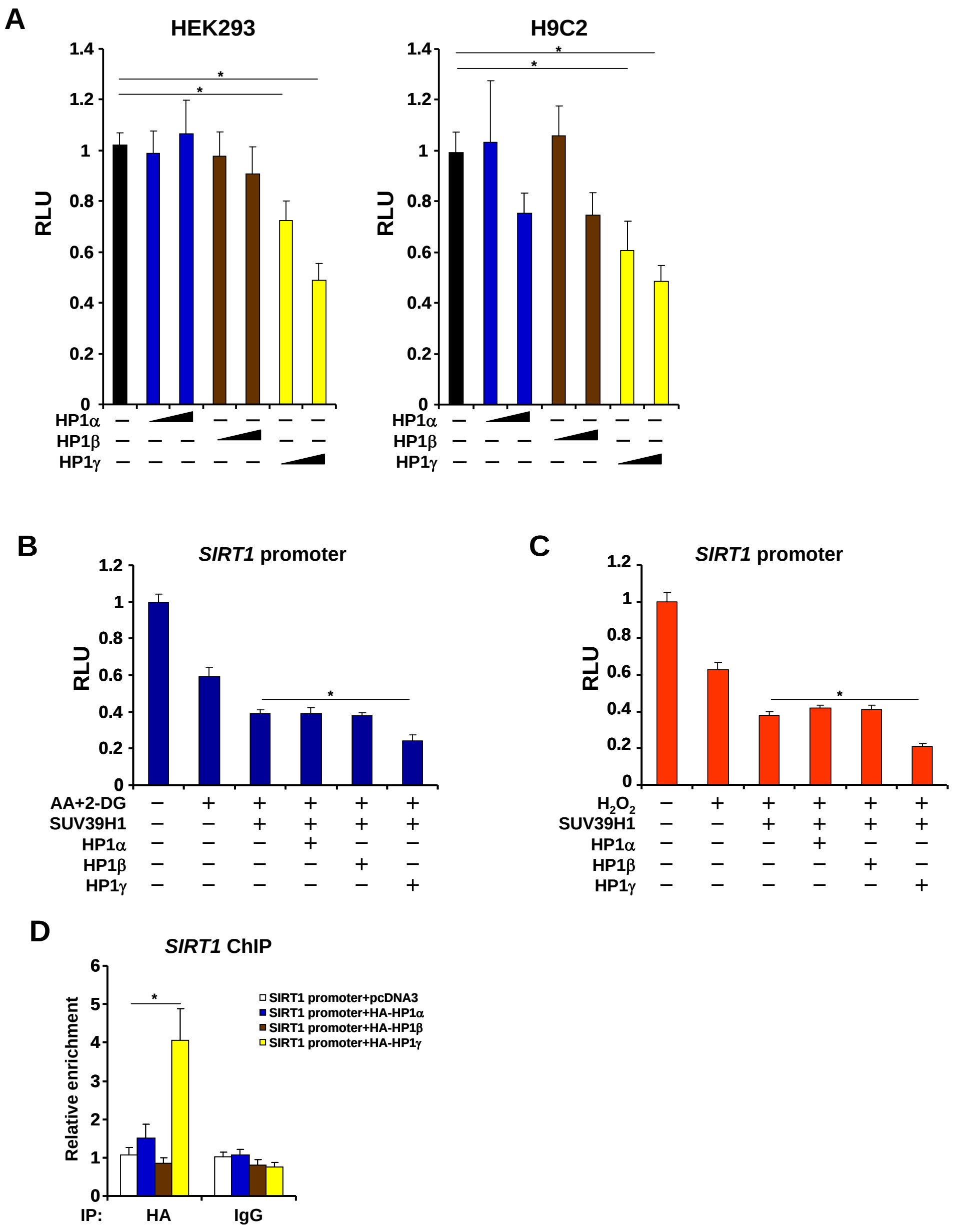

Supplementary Fig.16: ***HP1γ represses SIRT1 promoter activity.*** (**A**) A SIRT1 promoter construct(-115/+54) was transfected into HEK293 cells or H9C2 cells with indicated HP1 expression constructs. Luciferase activities were normalized by protein concentration and GFP fluorescence and expressed as relative luciferase unit (RLU). (**B, C**) A SIRT1 promoter construct (-115/+54) was transfected into H9C2 cells with indicated expression constructs followed by treatment with AA plus 2-DG (B) or H_2O_2 (C). Luciferase activities were normalized by protein concentration and GFP fluorescence and expressed as relative luciferase unit (RLU). (**D**) A SIRT1 promoter construct (-115/+54) was transfected into H9C2 cells with indicated expression constructs. ChIP assays were performed with indicated antibodies. Error bars represent standard deviation (N=3). *, $p < .05$ (One-way ANOVA with post-hoc Scheffe test).

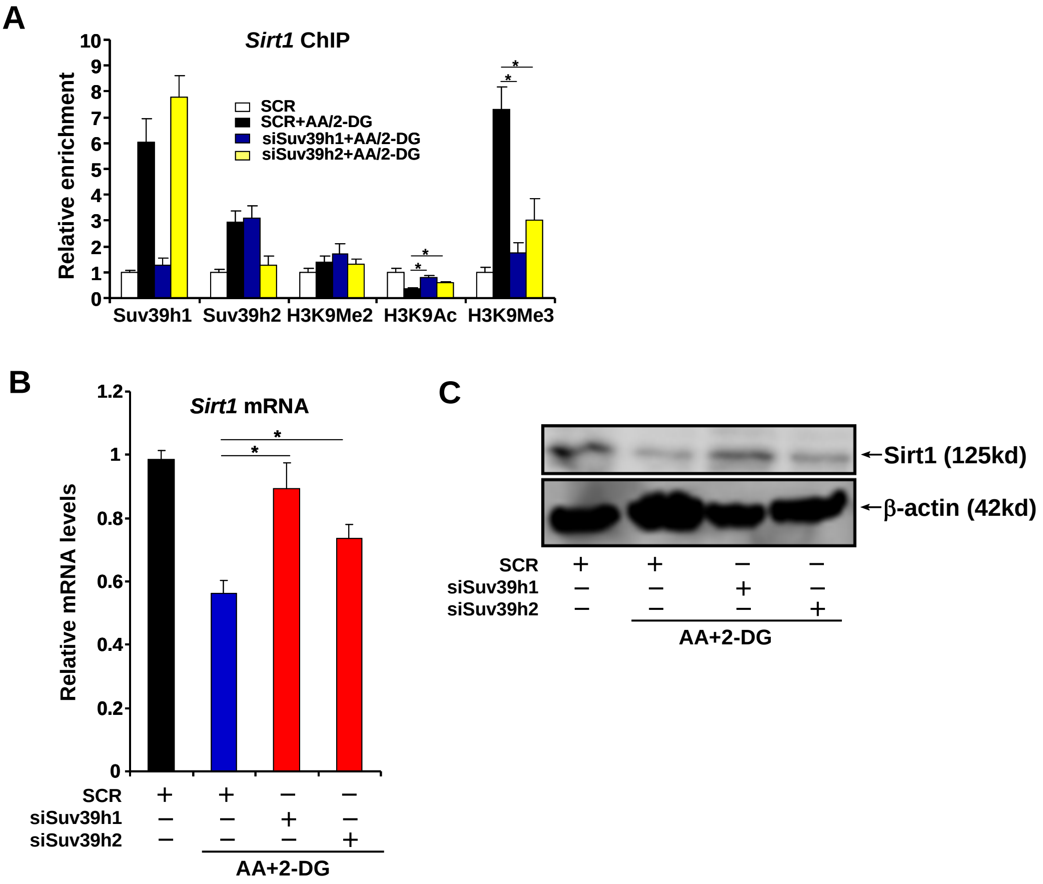

Supplementary Fig.17: ***SUV39H silencing antagonizes SIRT1 repression in AA+2-DG-treated H9C2 cells***. H9C2 cells were transfected with SUV39H siRNA or random siRNA (SCR) followed by treatment with AA+2-DG. ChIP assays were performed with indicated antibodies (A). SIRT1 expression was examined by qPCR (B) and Western (C). Error bars represent standard deviation (N=3). *, p＜.05 (One-way ANOVA with post-hoc Scheffe test).

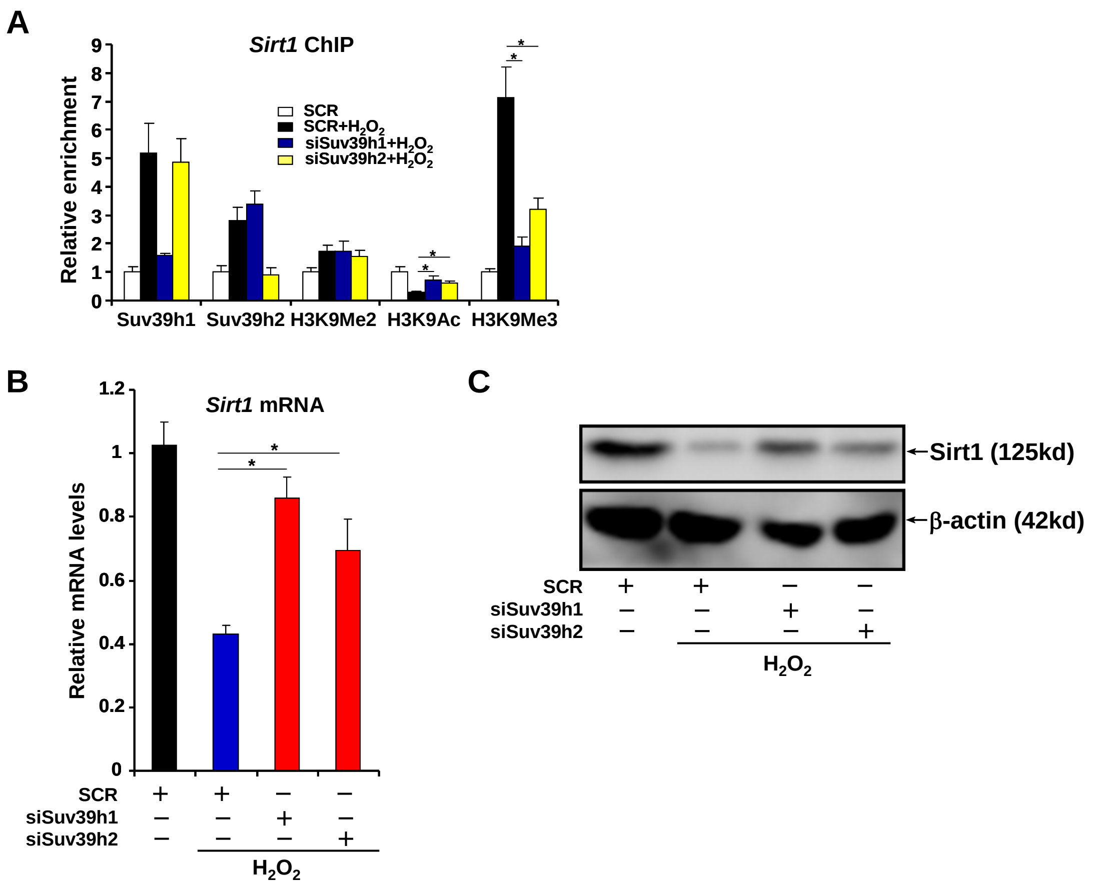

Supplementary Fig.18: ***SUV39H silencing antagonizes SIRT1 repression in H_2O_2-treated H9C2 cells***. H9C2 cells were transfected with SUV39H siRNA or random siRNA (SCR) followed by treatment with H_2O_2. ChIP assays were performed with indicated antibodies (A). SIRT1 expression was examined by qPCR (B) and Western (C). Error bars represent standard deviation (N=3). *, $p < .05$ (One-way ANOVA with post-hoc Scheffe test).

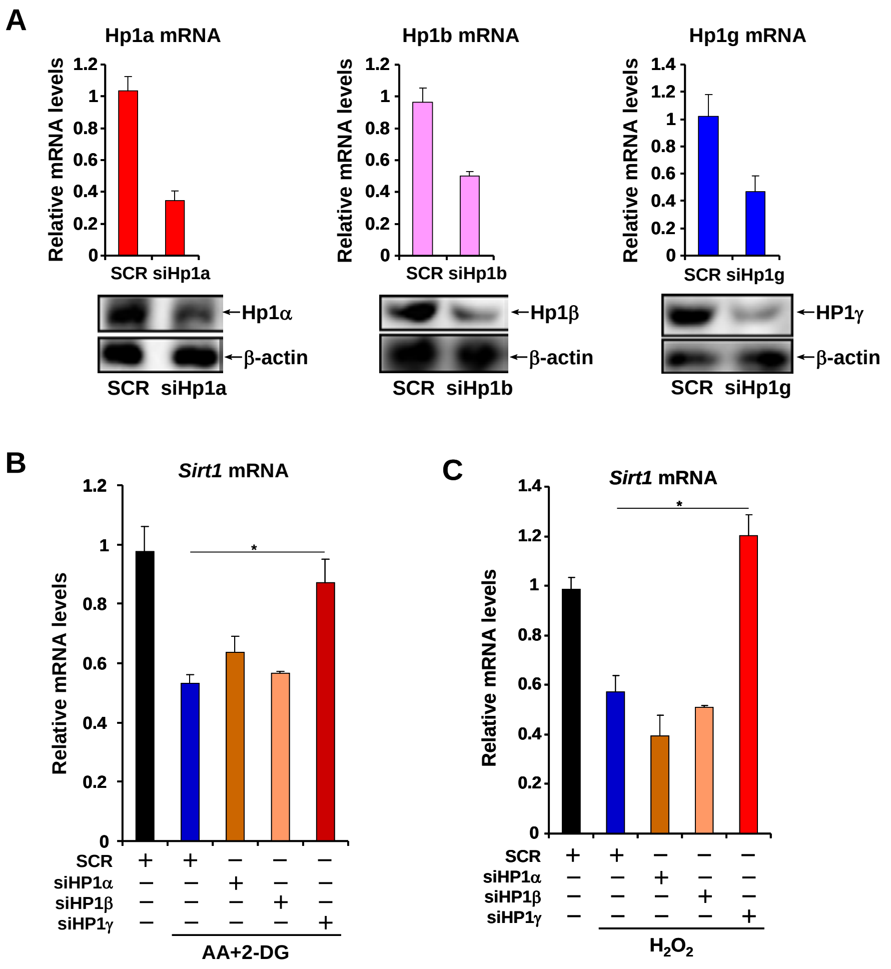

Supplementary Fig.19: *HP1γ silencing restores SIRT1 expression following ischemic/oxidative stress.* (**A**) Primary NRVMs were transfected with indicated HP1 siRNA. Knockdown efficiencies were verified by qPCR and Western. (**B, C**) Primary NRVMs were transfected with indicated HP1 siRNA followed by treatment with AA+2-DG (B) or H_2O_2 (C). SIRT1 expression levels were measured by qPCR. Error bars represent standard deviation (N=3). *, p<.05 (One-way ANOVA with post-hoc Scheffe test).

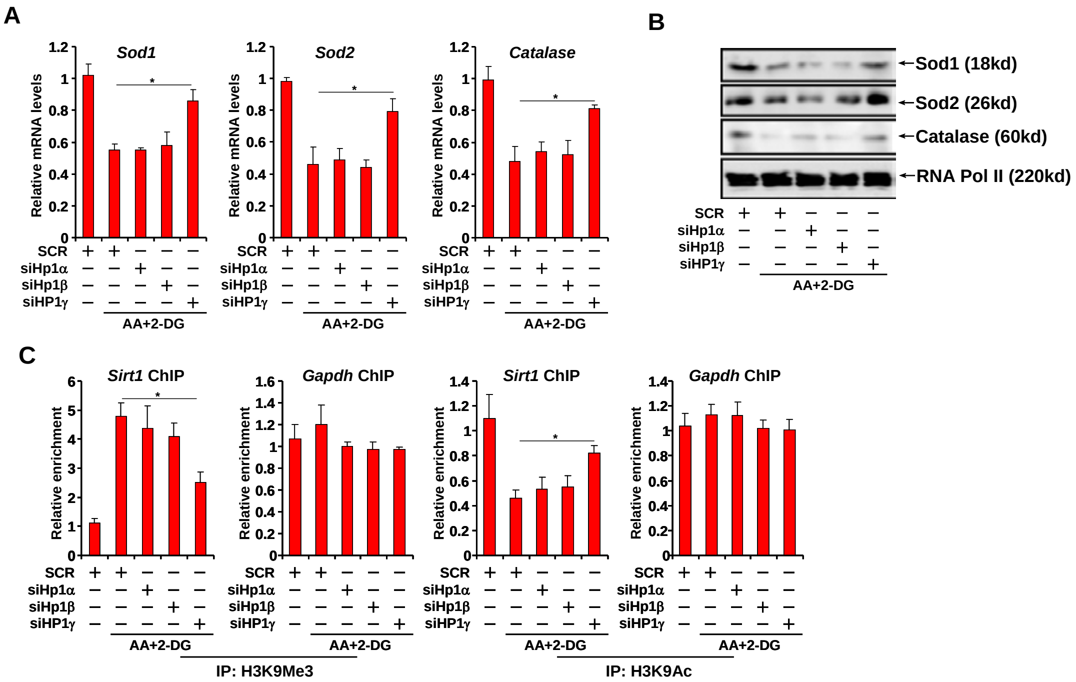

Supplementary Fig.20: ***HP1γ regulates the repression of anti-oxidant genes*.** (**A-C**) Primary NRVMs were transfected with indicated HP1 siRNA followed by treatment with AA+2-DG. Gene expression was measured by qPCR (A) and Western (B). ChIP assays were performed with indicated antibodies (C). Error bars represent standard deviation (N=3). *, $p < .05$ (One-way ANOVA with post-hoc Scheffe test).

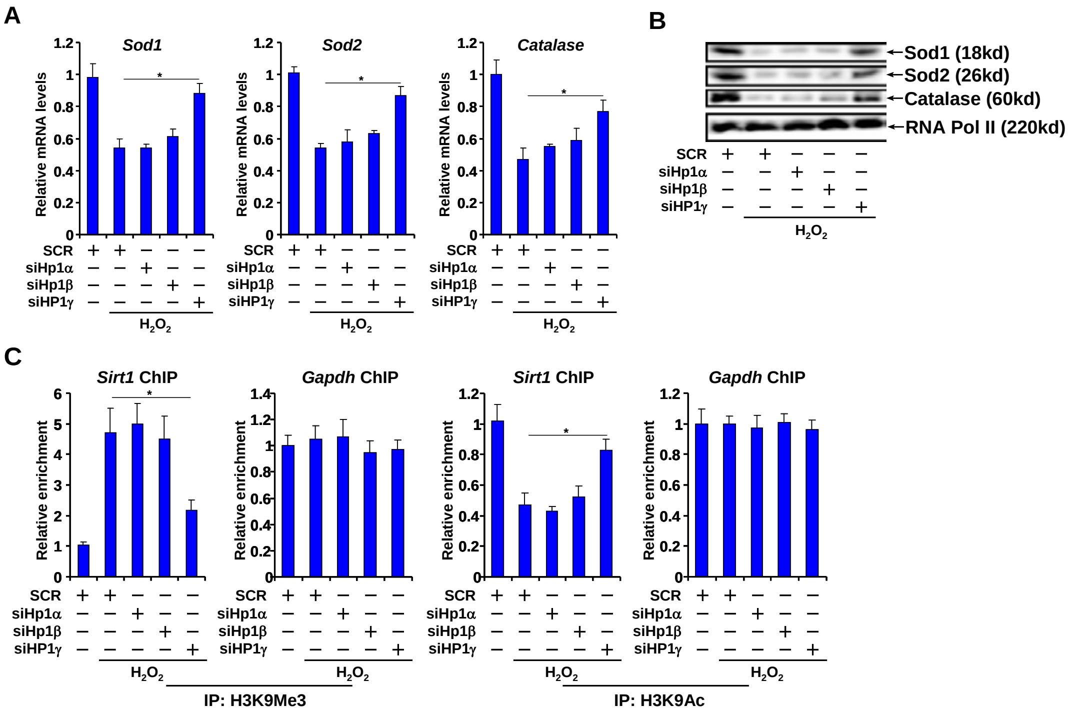

Supplementary Fig.21: ***HP1γ regulates the repression of anti-oxidant genes*.** (**A-C**) Primary NRVMs were transfected with indicated HP1 siRNA followed by treatment with H_2O_2. Gene expression was measured by qPCR (A) and Western (B). ChIP assays were performed with indicated antibodies (C). Error bars represent standard deviation (N=3). *, $p < .05$ (One-way ANOVA with post-hoc Scheffe test).

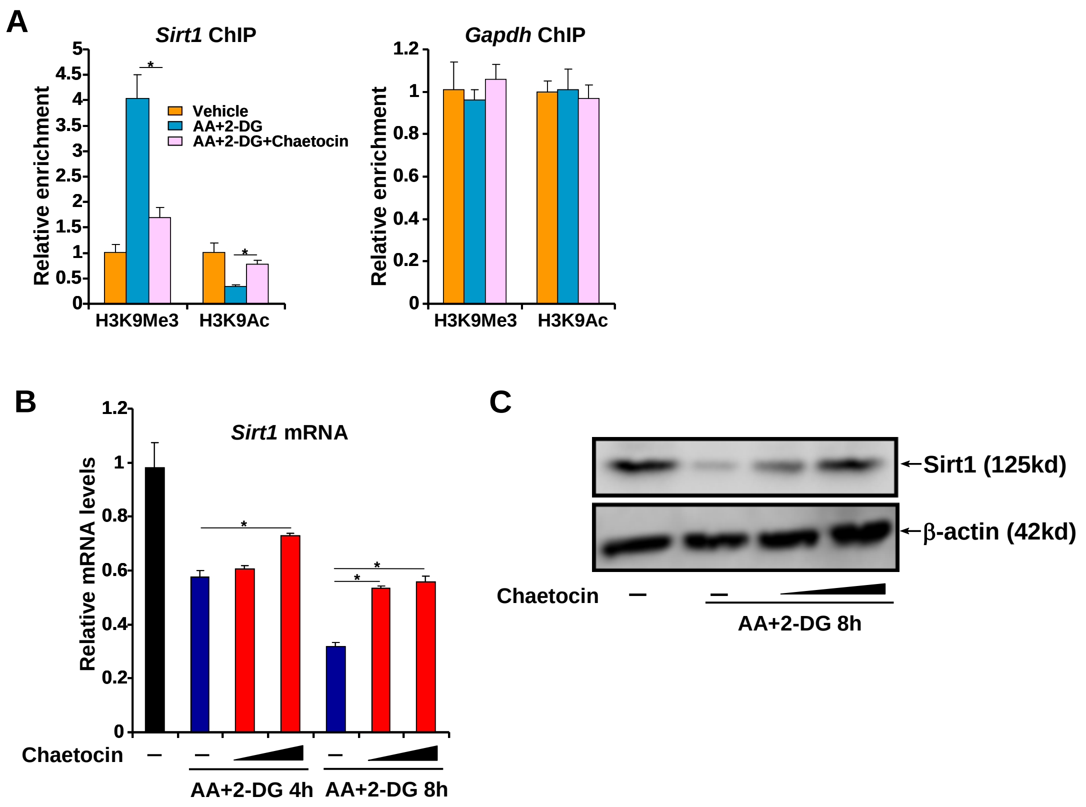

Supplementary Fig.22: ***Chaetocin antagonizes SIRT1 repression in AA+2-DG-treated H9C2 cells***. H9C2 cells were treated with AA+2-DG and chaetocin. (**A**) ChIP assays were performed with indicated antibodies. (**B**) SIRT1 mRNA expression was examined by qPCR. (**C**) SIRT1 protein expression was examined by Western. Error bars represent standard deviation (N=3). *, p＜.05 (One-way ANOVA with post-hoc Scheffe test).

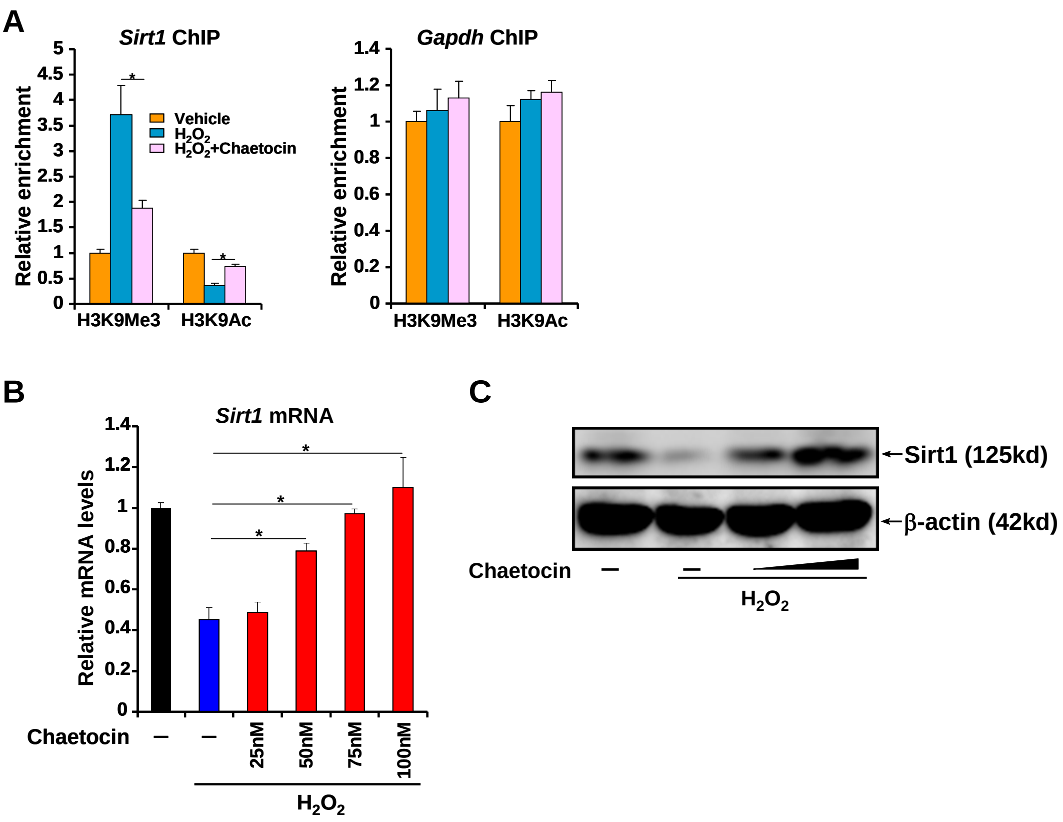

Supplementary Fig.23: ***Chaetocin antagonizes SIRT1 repression in H_2O_2-treated H9C2 cells***. H9C2 cells were treated with H_2O_2 and chaetocin. (**A**) ChIP assays were performed with indicated antibodies. (**B**) SIRT1 mRNA expression was examined by qPCR. (**C**) SIRT1 protein expression was examined by Western. Error bars represent standard deviation (N=3). *, $p < .05$ (One-way ANOVA with post-hoc Scheffe test).

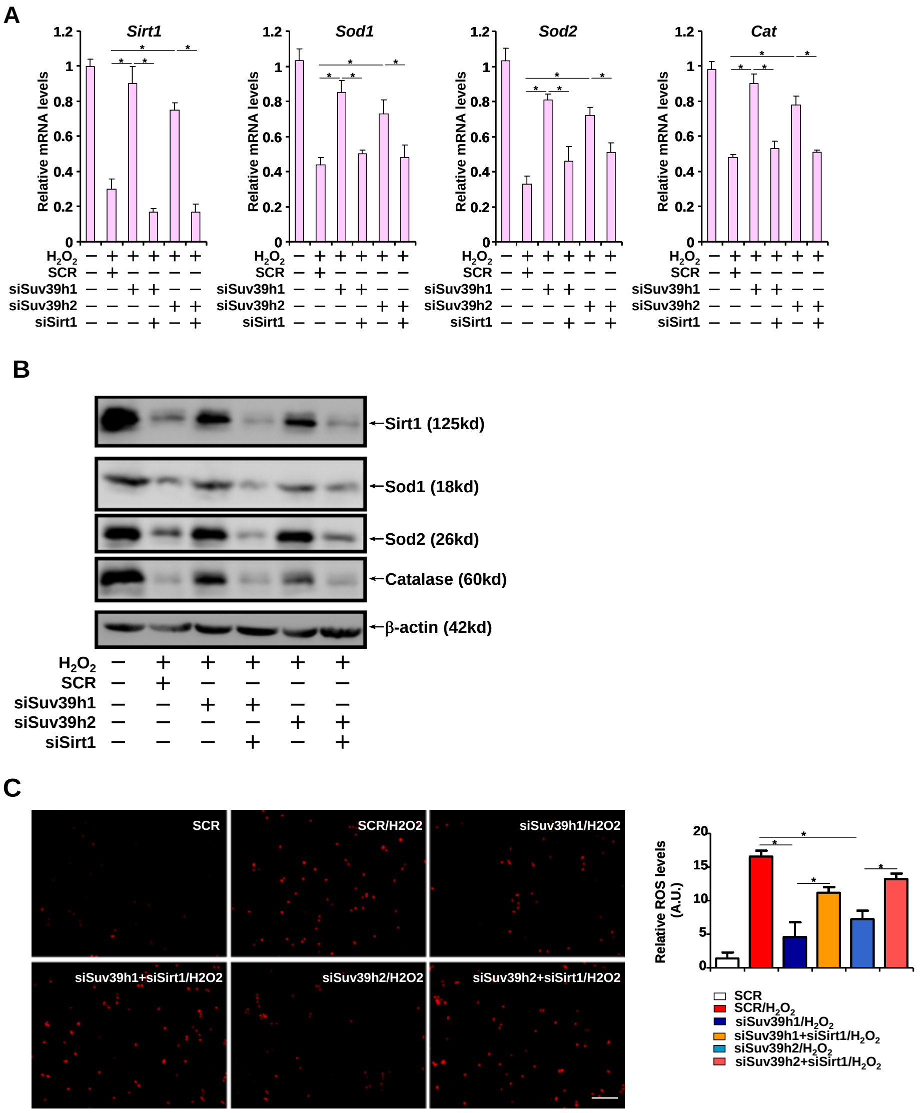

Supplementary Fig.24: *SUV39H regulates ROS production in a SIRT1-dependent manner in cardiomyocytes*. Primary NRVMs were transfected with siRNA targeting Suv39h1/h2, Sirt1, or scrambled siRNA (SCR) followed by treatment with H_2O_2 or EX-527. Expression levels of antioxidant genes were examined by qPCR (A) and Western (B). Intracellular ROS levels were evaluated by DHE staining (C). Scale bar, 20μm. Error bars represent standard deviation (N=3). *, p＜.05 (One-way ANOVA with post-hoc Scheffe test).

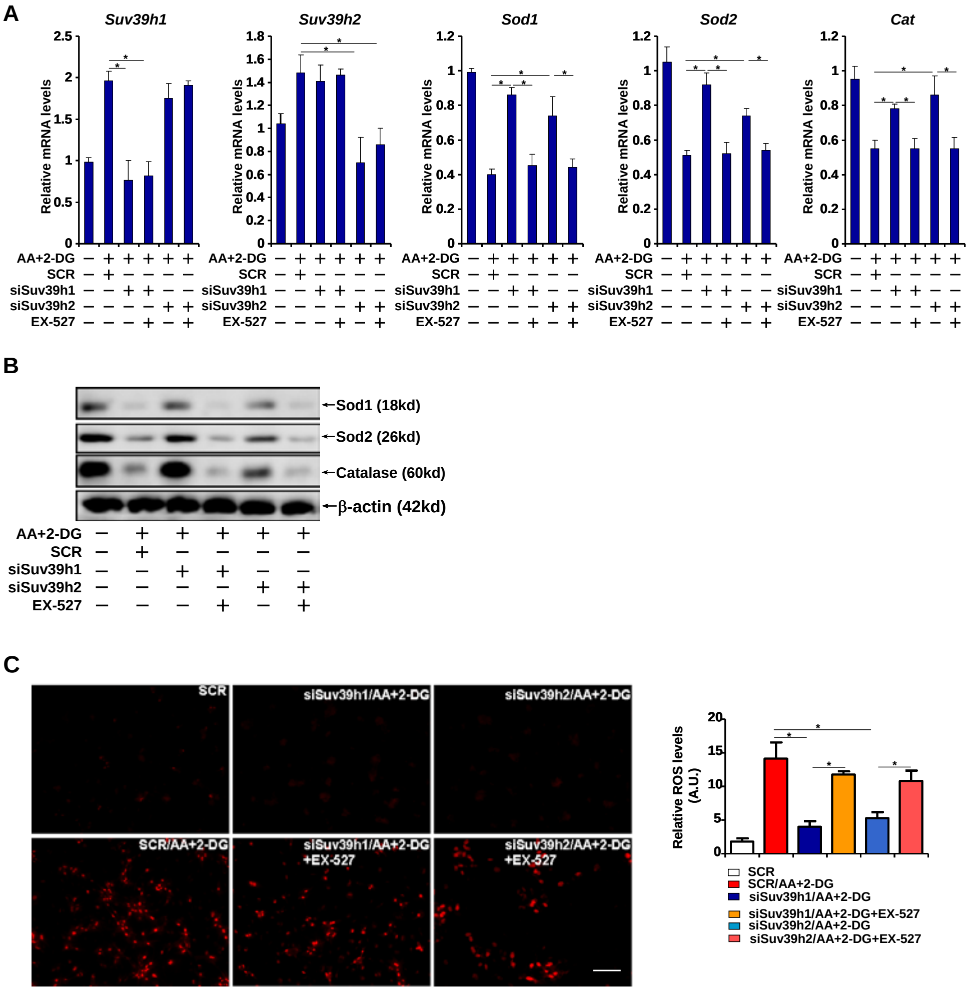

Supplementary Fig.25: ***SUV39H regulates ROS production in a SIRT1-dependent manner in cardiomyocytes***. Primary NRVMs were transfected with siRNA targeting Suv39h1/h2, Sirt1, or scrambled siRNA (SCR) followed by treatment with AA+2-DG or EX-527. Expression levels of antioxidant genes were examined by qPCR (A) and Western (B). Intracellular ROS levels were evaluated by DHE staining (C). Scale bar, 20μm. Error bars represent standard deviation (N=3). *, $p < .05$ (One-way ANOVA with post-hoc Scheffe test).

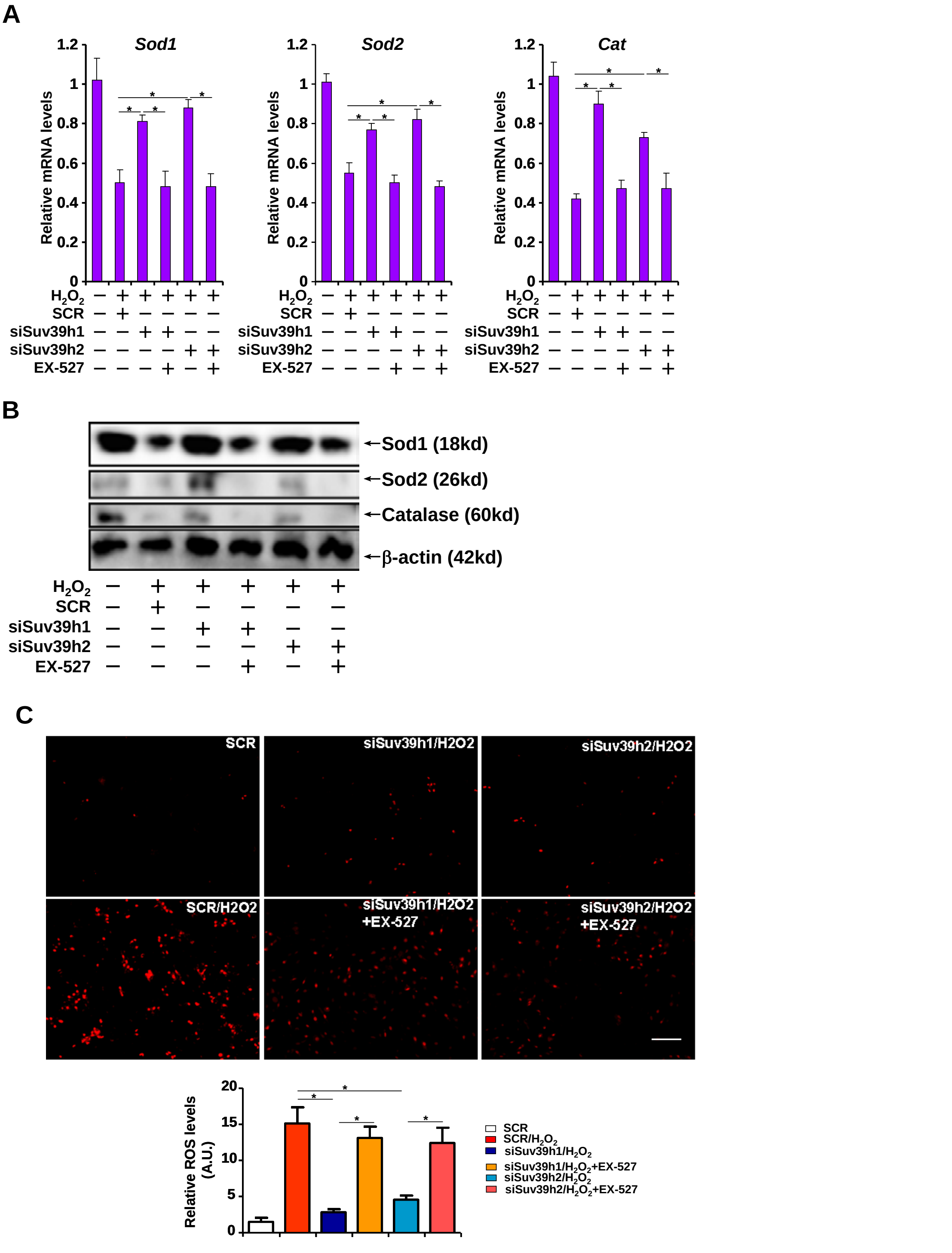

Supplementary Fig.26: ***SUV39H regulates ROS production in a SIRT1-dependent manner in cardiomyocytes***. Primary NRVMs were transfected with siRNA targeting Suv39h1/h2 or scrambled siRNA (SCR) followed by treatment with H2O2 or EX-527. Expression levels of antioxidant genes were examined by qPCR (A) and Western (B). Intracellular ROS levels were evaluated by DHE staining (C). Scale bar, 20μm. Error bars represent standard deviation (N=3). *, $p < .05$ (One-way ANOVA with post-hoc Scheffe test).

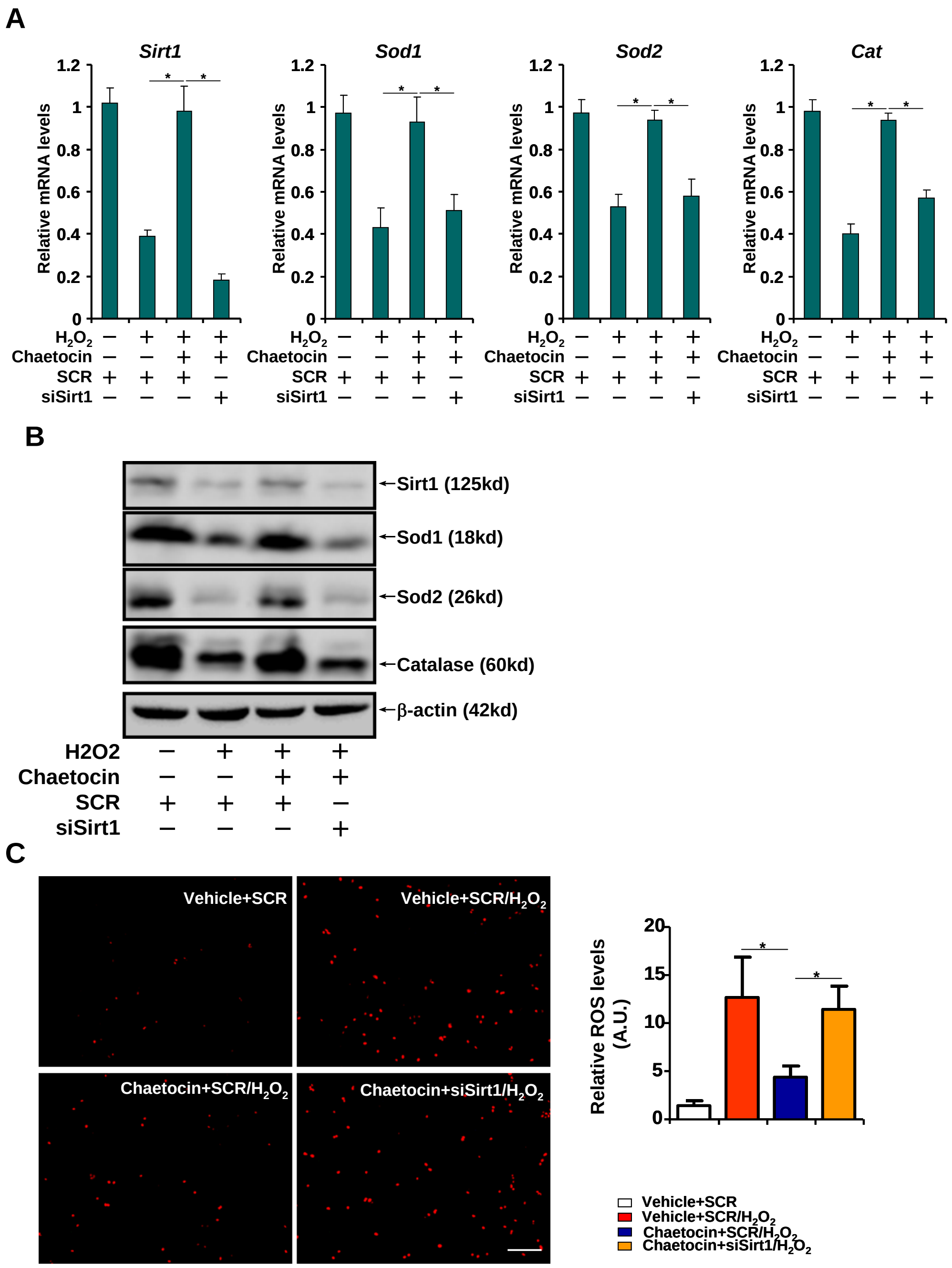

Supplementary Fig.27: *Chaetocin regulates ROS production in a SIRT1-dependent manner in cardiomyocytes*. Primary NRVMs were transfected with siRNA targeting Suv39h1/h2, Sirt1, or scrambled siRNA (SCR) followed by treatment with H_2O_2 and/or chaetocin. Expression levels of antioxidant genes were examined by qPCR (A) and Western (B). Intracellular ROS levels were evaluated by DHE staining (C). Scale bar, 20μm. Error bars represent standard deviation (N=3). *, $p < .05$ (One-way ANOVA with post-hoc Scheffe test).

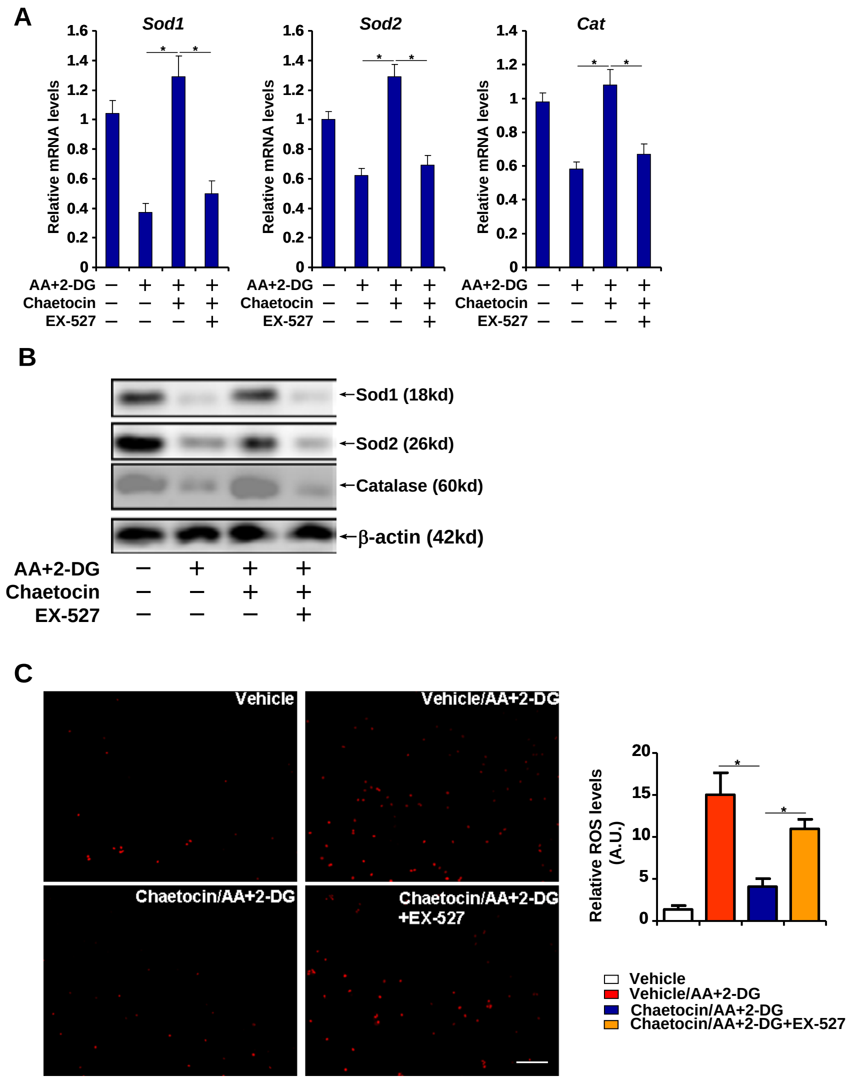

Supplementary Fig.28: ***Chaetocin regulates ROS production in a SIRT1-dependent manner in cardiomyocytes***. Primary NRVMs were treated with AA+2-DG, chaetocin or EX-527. Expression levels of antioxidant genes were examined by qPCR (A) and Western (B). Intracellular ROS levels were evaluated by DHE staining (C). Scale bar, 20μm. Error bars represent standard deviation (N=3). *, $p < .05$ (One-way ANOVA with post-hoc Scheffe test).

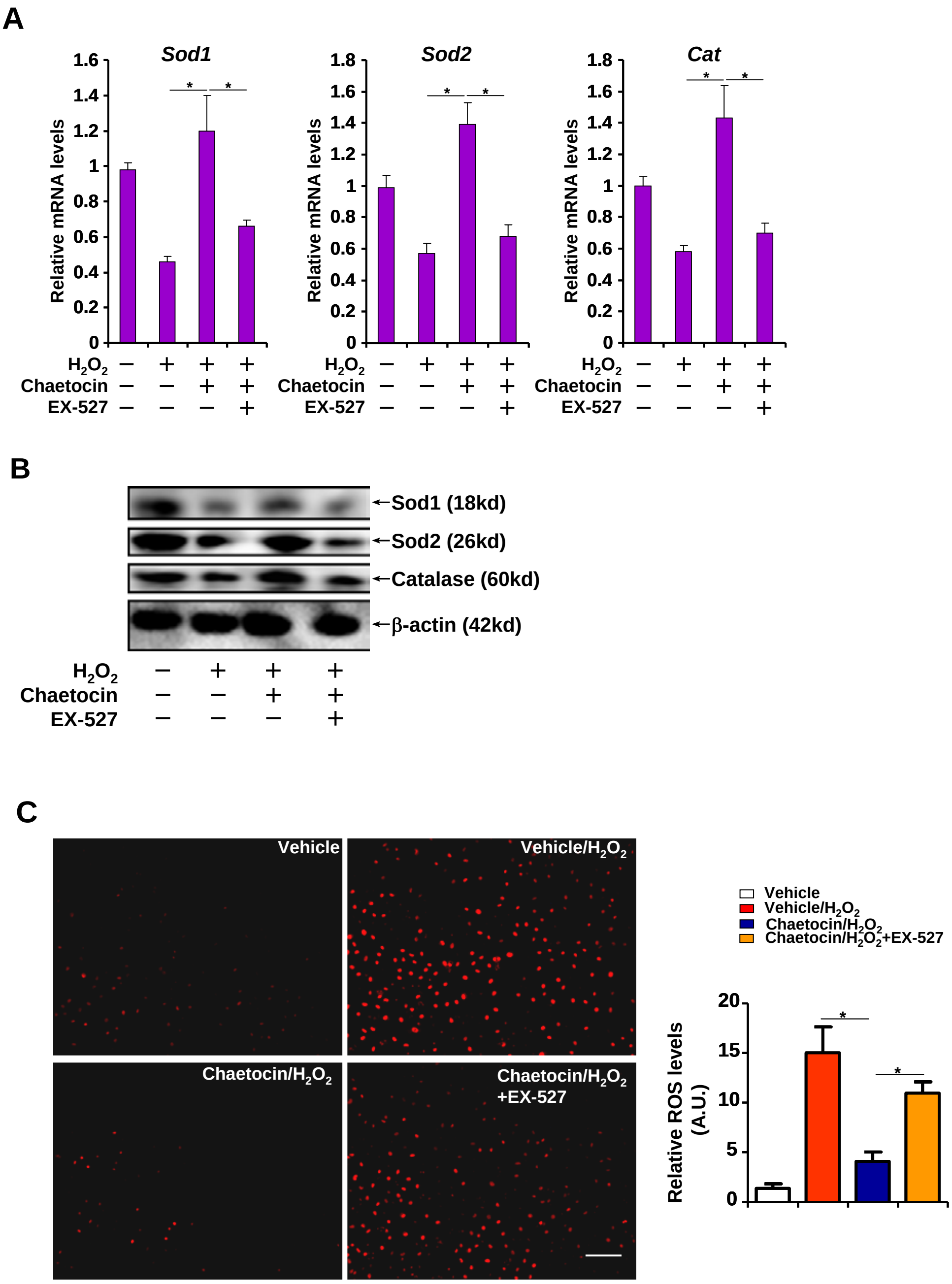

Supplementary Fig.29: ***Chaetocin regulates ROS production in a SIRT1-dependent manner in cardiomyocytes***. Primary NRVMs were treated with H_2O_2, chaetocin or EX-527. Expression levels of antioxidant genes were examined by qPCR (A) and Western (B). Intracellular ROS levels were evaluated by DHE staining (C). Scale bar, 20μm. Error bars represent standard deviation (N=3). *, $p < .05$ (One-way ANOVA with post-hoc Scheffe test).

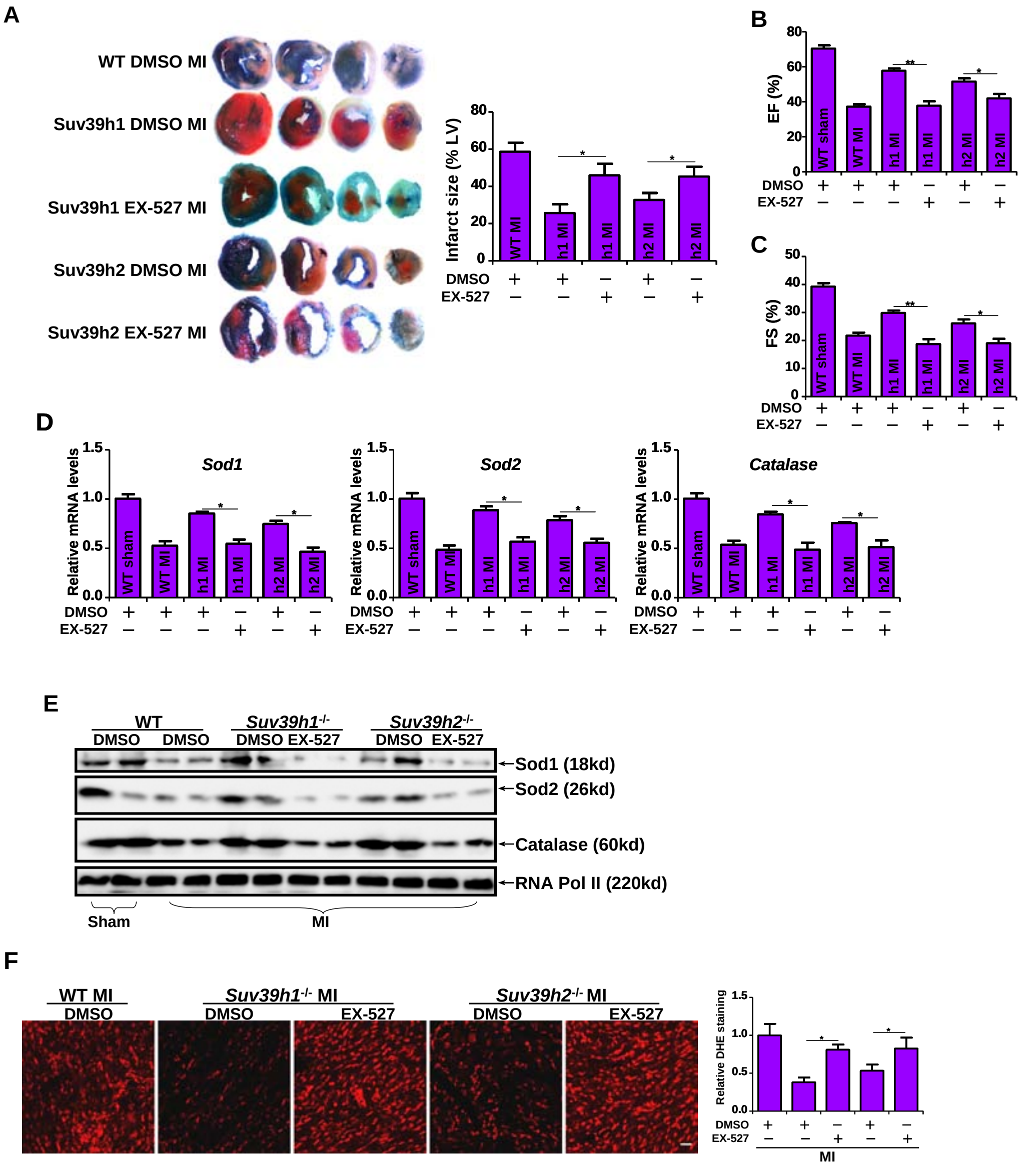

Supplementary Fig.30: ***SUV39H regulates myocardial infarction by targeting SIRT1 in mice*.** MI was induced in Suv39h1 knockout (*h1*$^{-/-}$) mice, Suv39h2 knockout (*h2*$^{-/-}$) mice or wide type (WT) littermates by LAD. Following the procedure, the mice were peritoneally injected with EX-527 (10mg/kg) daily till sacrifice. (A) Representative TTC staining. Infarct size was calculated and quantified by Image Pro. (B, C) EF and FS values were measured by echocardiography. (D, E) Expression levels of antioxidant genes were measured by qPCR (D) and Western (E). (F) Cardiac ROS levels were evaluated by DHE staining. Scale bar, 50μm. Error bars represent standard deviation (N=3 for the sham group and =5 for each of the MI groups). *, $p < .05$; **, $p < .01$ (One-way ANOVA with post-hoc Scheffe test).

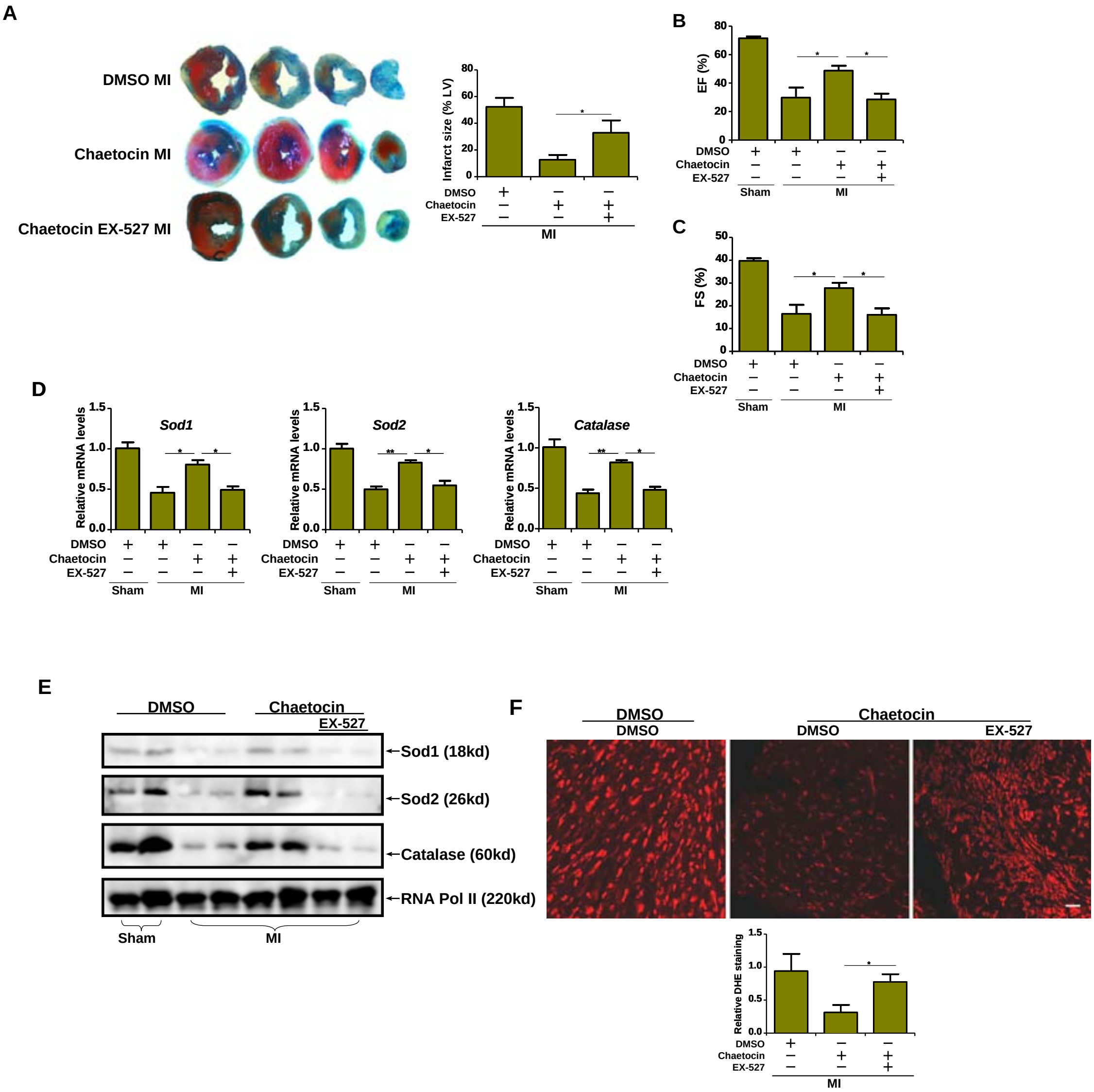

Supplementary Fig.31: ***Cardioprotective effects of chaetocin depends on SIRT1 in mice***. C57/BL mice were injected peritoneally with chaetocin (25mg/kg) or DMSO 2 days prior to the LAD procedure. Following the procedure, the mice were peritoneally injected with EX-527 (10mg/kg) daily till sacrifice. (A) Representative TTC staining. Infarct size was calculated and quantified by Image Pro. (B, C) EF and FS values were measured by echocardiography. (D, E) Expression levels of antioxidant genes were measured by qPCR (D) and Western (E). (F) Cardiac ROS levels were evaluated by DHE staining. Scale bar, 50µm. Error bars represent standard deviation (N=8 for each group). *, $p < .05$; **, $p < .01$ (One-way ANOVA with post-hoc Scheffe test).

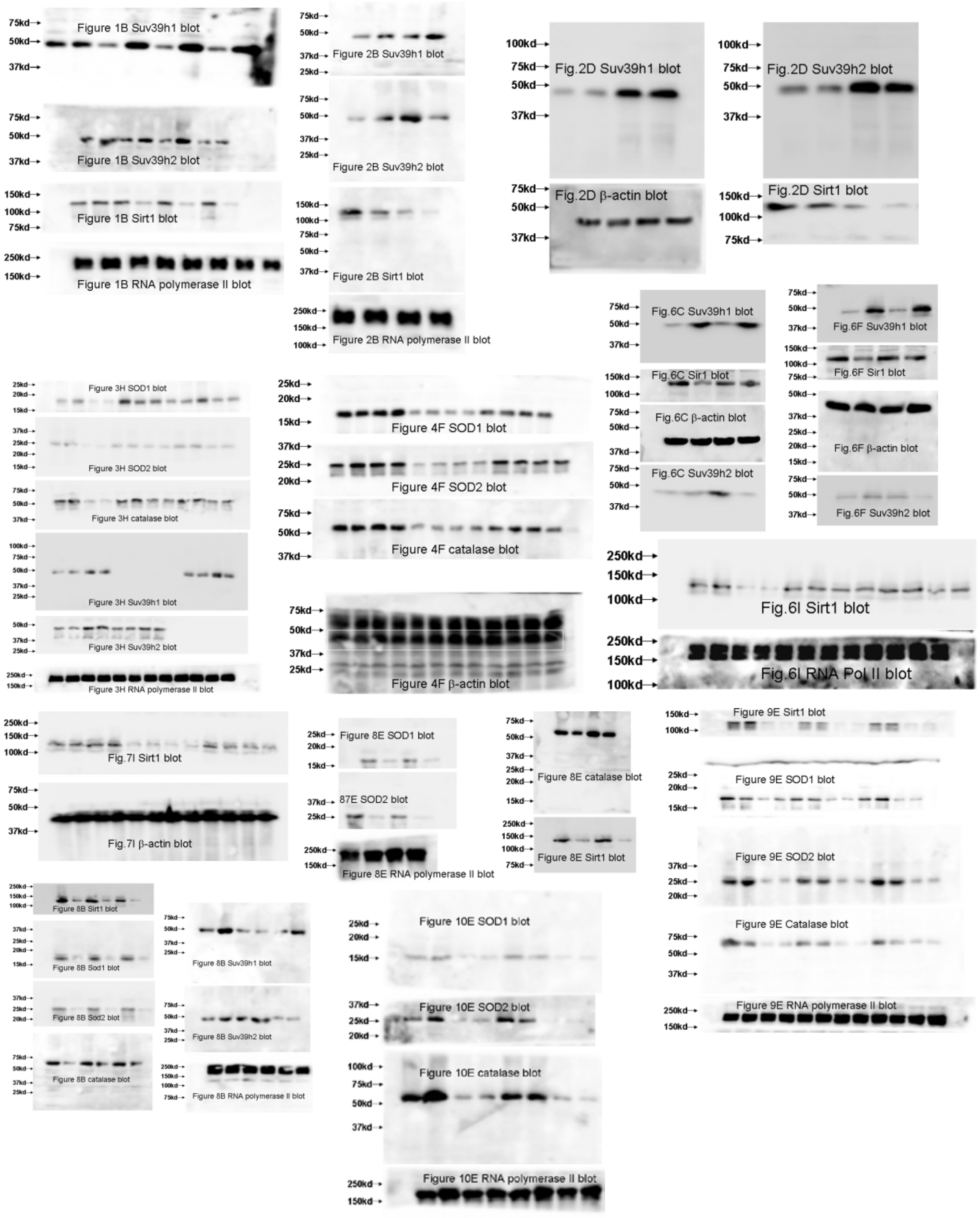

Supplementary Fig.32: Representative original un-cropped Western blots.

Supplementary Table 1: A summary of cardiac rupture (CR) incidents in different groups.

Genotype (N)	WT (20)	*Suv39h1*$^{-/-}$ (25)	*Suv39h2*$^{-/-}$ (28)
Cardiac rupture	5	1	3
Chi Test	*Suv39h1*$^{-/-}$ vs WT, p=.0349; *Suv39h2*$^{-/-}$ vs WT, p=.0455; *Suv39h1*$^{-/-}$ vs *Suv39h2*$^{-/-}$, p=.0368		

MI was induced in *Suv39h1* knockout mice, *Suv39h2* knockout mice, or wide type (WT) littermates by LAD.
